# Selective vulnerability and resilience of thalamic neuronal populations in Frontotemporal Dementia

**DOI:** 10.21203/rs.3.rs-10670822/v1

**Published:** 2026-09-11

**Authors:** Amber E. Trujillo, Mira Sohn, Magdalena J. Macias, Martyna M. Grochowska, John Joyce, Isabelle Leber, Dann Huh, Anton Schulmann, Francis J. McMahon, Kendall Van Keuren-Jensen, Morwena Latouche, Elise Marsan

**Affiliations:** 1- Molecular Pathology Unit, Center for Alzheimer’s and Related Dementias, National Institute on Aging, National Institutes of Health, Bethesda, MD 20892, USA; 2- Data Tecnica International LLC, Washington DC, USA; 3- Research, Biogen Inc, Cambridge, MA 02142, USA; 4- Ecole Pratique des Hautes Etudes, PSL Research University, 75014 Paris, France.; 5- Paris Brain Institute (ICM), Inserm U1127, CNRS UMR 7225, Sorbonne University, APHP-Hôpital Pitié-Salpêtrière, Paris 75013, France; 6- Department of Psychiatry, Columbia University; New York State Psychiatric Institute, New York, NY 10032, USA; 7- Human Genetics Branch, National Institute of Mental Health, National Institutes of Health, Bethesda, MD 20892, USA; 8- Bioinnovation and Genome Sciences Division, Translational Genomics Research Institute (TGen), City of Hope, Phoenix, AZ 85004, USA

## Abstract

Frontotemporal dementia (FTD) caused by progranulin (*GRN*) mutations is associated with pronounced thalamic degeneration, yet the cellular basis of selective thalamic vulnerability remains poorly understood. Here, we generated an integrated single-nucleus RNA-sequencing atlas of the human antero-median thalamus comprising 331,065 nuclei from 92 individuals across three independent cohorts spanning *GRN*-associated FTD, sporadic FTD with TDP-43 proteinopathy, Alzheimer’s disease (AD), and neurologically normal aging. While AD samples exhibited minimal thalamic cellular remodeling, FTD cases were characterized by highly selective and reproducible degeneration of discrete neuronal populations across independent cohorts. In particular, two inhibitory neuronal populations were markedly depleted in FTD, which was accompanied by impaired GABAergic communication. Among excitatory neurons, *RNF220*+ neurons exhibited extensive transcriptional dysregulation, altered sterol metabolic pathways, together with robust TDP-43-associated *STMN2* cryptic splicing pathology and reduced glutamatergic signaling. By contrast, *RNF220*− excitatory neurons were comparatively resilient, displaying preserved neuronal communication, and resistance to *STMN2* cryptic exon accumulation. In control individuals, *RNF220*-excitatory neurons exhibited elevated *GRN* expression and an intrinsic enrichment of cytoskeletal maintenance programs. Spatial transcriptomics confirmed the anatomical distribution of vulnerable neuronal populations in the adult human thalamus. In parallel, FTD samples demonstrated expansion of vascular-associated and lymphocyte populations as well as reactive glial signatures. Together, these findings define a cellular framework for selective vulnerability and resilience in the human FTD thalamus and identify intrinsic molecular programs associated with resistance to neurodegeneration.

## Introduction:

Frontotemporal dementia (FTD) is a neurodegenerative disease characterized by progressive behavioral, cognitive, and language impairment driven by the degeneration of frontal and temporal brain networks (Grossman et al., 2023). Although substantial progress has been made in defining the cortical pathology underlying FTD, the selective vulnerability of subcortical circuits and their contribution to disease progression remains incompletely understood. Among these structures, the thalamus has emerged as a critical hub implicated in the early cognitive, behavioral, and affective dysfunction. This vulnerability is shared across multiple forms of FTD (Bocchetta et al., 2018; Soskic et al., 2025), including sporadic FTD with TDP-43 proteinopathy (sFTD-TDP) and familial FTD-TDP caused by heterozygous loss-of-function mutations in *GRN* (FTD-*GRN*) (Baker et al., 2006; Cruts et al., 2006).

Experimental studies in progranulin-deficient mouse models further support a central role for the thalamus in FTD pathogenesis. Advanced aging in these models consistently reproduces robust thalamic neurodegeneration (Frew & Nygaard, 2021; Nader et al., 2025; Yin et al., 2010). Crucially, this structural loss is accompanied or preceded by profound network-level impairment, characterized by aberrant microglial pruning of inhibitory synapses and thalamo-cortical circuits hyperexcitability (Lui et al., 2016; Zhang et al., 2020).

Consistent with these findings, human neuroimaging studies in presymptomatic *GRN* mutation carriers revealed thalamo-cortical hyperconnectivity (Lee et al., 2019), a prodromal network alteration that precedes the severe structural thalamic atrophy characteristic of symptomatic stages. At the cellular level, mutations in *GRN* disrupt essential lysosomal degradation pathways and lipid homeostasis, reflecting progranulin’s pleiotropic role as a critical regulator of endolysosomal function and cellular maintenance (Kao et al., 2017). In addition to these organelle-level defects, *GRN* loss of function is strongly linked to a secondary loss of functional nuclear TDP-43 (Cairns et al., 2007; Marsan et al., 2023; Ramsey et al., 2026), driving the pathogenic inclusion of cryptic exons in critical downstream targets such as *STMN2* (de Majo et al., 2023; Klim et al., 2019; Ling et al., 2015; Melamed et al., 2019). However, because progranulin is ubiquitously expressed, the precise cell-type-specific mechanisms that translate these localized lysosomal and lipid defects into early network hyperconnectivity and subsequent structural degeneration within human thalamic circuits remain unknown.

Recent transcriptomic and neuropathological studies have identified disease-associated alterations within the FTD-*GRN* thalamus, including glial pathology and neurodegeneration in one FTD-*GRN* cohort (Marsan et al., 2023). However, cross-cohort characterization of the human thalamus in FTD is missing, as previous single-nuclei transcriptomic studies of FTD have focused on cortical regions (Belchikov et al., 2025; Gerrits et al., 2022; Gittings et al., 2023; Li et al., 2023; Natarajan et al., 2021; Pineda et al., 2024). While Alzheimer’s disease (AD) has also been associated with thalamic alterations (Mathys et al., 2024), the extent of cellular remodeling observed in AD appears substantially less pronounced than in FTD, providing an opportunity to distinguish disease-specific from more general neurodegenerative processes.

Here, we present an integrated single-nucleus RNA-sequencing (snRNA-seq) atlas of the human rostral to mid-thalamus across FTD-*GRN*, sFTD-TDP, AD, and neurologically normal aging using three independent human cohorts. By combining a newly generated FTD patient cohort with published human single-cell datasets, we established a rigorous cohort comparison framework enabling discrimination of disease-specific versus cohort-specific cellular alterations. Cross-cohort analysis identified reproducible and highly selective vulnerability of discrete inhibitory and excitatory neuronal populations in FTD, while AD samples exhibited minimal thalamic cellular remodeling. By using spatial transcriptomics, differential expression analysis, cell-cell communication modeling, and cryptic splicing analysis, we define molecular programs associated with both neuronal degeneration and resilience. We reveal marked depletion of discrete inhibitory interneuron subtypes and a subsequent decrease of GABAergic communication. Within excitatory circuits, we show that vulnerable *RNF220*+ neurons exhibit severe transcriptional dysregulation, altered sterol metabolism, and robust *STMN2* cryptic splicing pathology. Conversely, neighboring *RNF220*− excitatory neurons remain remarkably resilient, displaying baseline elevation of *GRN* expression and resistance to *STMN2* cryptic exon accumulation. Combined with a coordinated expansion of vascular and neuroimmune populations, these findings provide a single-cell blueprint of the human thalamus in FTD, uncovering cell-type-specific mechanisms governing selective vulnerability and resilience in FTD.

## Results:

### 1. Single-cell profiling reveals disease-specific thalamic remodeling in FTD

Thalamic dysfunction is increasingly implicated in the cognitive and behavioral deficits associated with frontotemporal dementia (FTD), yet the cellular architecture underlying selective thalamic vulnerability in human disease remains poorly defined. To systematically characterize thalamic cell states across neurodegeneration, we generated an integrated single-nucleus RNA-sequencing (snRNA-seq) atlas comprising 331,065 nuclei from 92 individuals spanning progranulin-associated FTD (FTD-*GRN*), sporadic FTD with TDP-43 proteinopathy (sFTD-TDP), Alzheimer’s disease (AD), and age- and sex-matched controls across three independent cohorts (Figure 1A–C; Supplementary Figure 1; **Supplementary Table 1**).

All major thalamic cell classes, including astrocytes, oligodendrocytes, oligodendrocyte precursor cells, microglia, neurons, vascular-associated cells, and lymphocyte populations, were identified based on canonical marker gene expression across cohorts (Figure 1D–F, **Supplementary Table 2**). Comparative analysis of cellular composition revealed marked disease-specific remodeling in all FTD cases, characterized by significant neuronal depletion accompanied by expansion of vascular-associated and lymphocyte populations, whereas overall cellular composition remained largely preserved in AD relative to controls (Figure 1G). When stratifying by disease etiology, we observed that the reduction in neuronal proportions and expansion of glial and vascular-associated populations were consistent in *FTD-GRN* mutation carriers. A similar pattern of cellular remodeling was evident in sFTD-TDP cases (Supplementary Figure 2B); however, while these trends mirrored the FTD-*GRN* cases, they did not reach statistical significance, likely reflecting the limited sample size of this subgroup.

Because progranulin deficiency is a primary driver of FTD-*GRN* pathogenesis, we next examined *GRN* expression across thalamic cell populations in control individuals. Relative *GRN* expression (log-CP10K) varied substantially across major cell classes and was enriched within microglia and blood vessel-associated populations, while remaining comparatively consistent across other cell types, including pan-neuronal populations (Figure 1H,I). These findings suggest that cell-type-specific progranulin expression alone does not fully explain selective neuronal vulnerability in the FTD thalamus, prompting further investigation of molecular heterogeneity across neuronal subtypes.

### 2. Molecular taxonomy of thalamic neuronal populations

To define the neuronal populations underlying selective thalamic vulnerability, we subclustered thalamic neurons and identified four major neuronal populations composed of two excitatory and two inhibitory neuronal subtypes (Figure 2A–C). Cell-type annotation was guided by previously reported human thalamic transcriptional signatures (Bakken et al., 2021; Marsan et al., 2023; Mathys et al., 2024; Siletti et al., 2023), and refined using the large scale and cross-cohort structure of our integrated dataset. These neuronal populations were distinguished by robust transcriptional signatures, including pan-inhibitory expression of *GAD2* and *MEIS2* together with differential expression of *SOX14*, *NR2F2*, *ERBB4*, *LAMP5*, and *BCL11B* across inhibitory neuronal populations. While InNeu *SOX14*+ neurons correspond to midbrain-derived interneurons, the InNeu *SOX14*-population is composed of ganglionic eminence-derived subtypes, encompassing medial ganglionic eminence (MGE; marked by *LHX6*, *SST*, and *NPY*) and caudal ganglionic eminence (CGE; marked by *CXCL14, VIP,* and *LAMP5*) lineages (Supplementary Figure 2A). In contrast, excitatory neuronal populations were defined by *SLC17A6* expression, consistent with thalamic projection neuron identity, and differential *RNF220* expression (Figure 2A,B).

We therefore annotated these populations as InNeu *SOX14*+, InNeu *SOX14*−, ExNeu *RNF220*+, and ExNeu *RNF220*− (Figure 2C). Integration with human spatial transcriptomic datasets generated by Schulmann et al. (Schulmann et al., 2024) (Schulmann et al. *under revision*) showed that InNeu *SOX14*+ and ExNeu *RNF220*+ populations were present across several nuclei in the human medial thalamus (Figure 2D).

Because selective vulnerability in FTD may reflect intrinsic differences in progranulin biology, we next examined relative *GRN* expression across neuronal subtypes in control individuals. Notably, *GRN* expression was highest in the ExNeu *RNF220*− population, whereas the remaining neuronal populations exhibited comparatively lower expression levels (Figure 2E,F). These findings raised the possibility that thalamic neuronal populations exhibit differential susceptibility to neurodegeneration based on intrinsic molecular programs associated with progranulin expression.

### 3. Selective vulnerability of inhibitory and excitatory neuronal populations in FTD

To determine whether specific thalamic neuronal populations exhibit selective vulnerability in neurodegeneration, we performed differential abundance analysis across neuronal subtypes. Both inhibitory neurons (InNeu), InNeu *SOX14*+ and InNeu *SOX14*−, were significantly depleted in FTD relative to controls, indicating pronounced vulnerability of inhibitory thalamic circuits (Figure 3A). Stratification by disease etiology confirmed that this inhibitory loss was statistically significant in FTD-*GRN* individuals (Supplementary Figure 2C), while a similar trend toward depletion was observed in sporadic FTD-TDP cases, which did not reach statistical significance, likely due to the smaller cohort size (Supplementary Figure 2D). Immunohistochemical analysis confirmed reduced abundance of GAD65+ pan inhibitory neurons in FTD-*GRN* tissue (Figure 3B).

Prior to differential gene expression analyses in FTD, we applied RUVSeq-based correction (Risso et al., 2014) to remove residual study-specific transcriptional variation not fully captured by cohort as a fixed covariate in the generalized linear model. This approach minimized potential cohort- and chemistry-specific effects arising from the integration of two independent single-nucleus datasets, substantially reducing cohort-driven structure while preserving biological signals of interest (Supplementary Figure 3) and supporting the robustness of downstream cross-cohort comparisons. Within inhibitory neuronal populations, differential expression analysis identified relatively modest transcriptional alterations. Notably, InNeu *SOX14*− neurons exhibited reduced expression of *KCNAB1*, a voltage-gated potassium channel regulatory subunit involved in neuronal excitability, consistent with impaired inhibitory neuronal function preceding or accompanying neuronal loss (Figure 3E, **Supplementary Table 3**). Beyond *KCNAB1*, only a limited number of genes were differentially expressed in surviving inhibitory neurons, suggesting minimal transcriptional remodeling despite pronounced reductions in cell abundance (Figure 3 A–E), though the reduced cell numbers may limit statistical power and contribute to the attenuated transcriptomic signal. Together, these findings indicate that inhibitory neuronal vulnerability in FTD is associated with surprisingly limited molecular adaptation in the remaining cells, raising the possibility that these populations either undergo rapid degeneration or are affected by disease processes that precede the terminal stages captured in postmortem tissue.

In contrast to the broad depletion observed in inhibitory neurons, excitatory neurons (ExNeu) exhibited divergent molecular responses to disease. Immunohistochemical analysis revealed disrupted neuronal arborization within excitatory neuronal populations in FTD-*GRN* tissue (Figure 3B). Differential expression analysis demonstrated substantially greater transcriptional dysregulation within ExNeu *RNF220*+ neurons relative to other neuronal populations (Figure 3C–G), suggesting that this population represents a highly vulnerable excitatory neuronal subtype. Pathway enrichment analysis of dysregulated genes in ExNeu RNF220+ neurons revealed significant upregulation of sterol and cholesterol biosynthetic pathways, including *SQLE* and *MSMO1*, together with genes involved in cholesterol sensing and trafficking such as *INSIG1*, *LDLR*, and *NPC1* (Figure 3F,H; **Supplementary Tables 3 and 4**). Beyond metabolic adaptations, transcriptional shifts highlighted extensive neuron projection and cytoskeletal organization, marked by the downregulation of projection inhibitors (*SEMA3F*, *NOG*, *RAB29*) and the concurrent upregulation of active actin machinery (*ROCK1*, *PIK3CA*). This internal remodeling was mirrored externally by an enrichment in external adhesion and synapse stress pathways, driven by the upregulation of matrix hooks (*ITGAV*, *ITGB8*), the presynaptic scaffold *CASK*, and the synapse-elimination marker *B2M*. This coordinated signature of metabolic dysregulation, architectural reshaping, and cell-surface detachment underscores a profound structural and cellular instability that highlights the selective vulnerability of this excitatory population.

By contrast, ExNeu *RNF220*− neurons were largely transcriptionally preserved, with *ATP2B1* representing the only significantly differentially expressed gene detected in FTD relative to controls (Figure 3G). This striking preservation contrasts with the extensive transcriptional remodeling observed in ExNeu *RNF220*+ neurons and supports the existence of a highly resilient excitatory neuronal population within the FTD thalamus. Notably, ExNeu *RNF220*− neurons also exhibited the highest baseline *GRN* expression among thalamic neuronal subtypes in controls (Figure 2E), as well as in individuals with AD and FTD (Figure 3I), whether in sporadic FTD-TDP or FTD-*GRN* (Supplementary Figure 2E). This pattern was maintained despite the overall reduction in *GRN* expression across all neuronal subtypes associated with *GRN* mutations. These findings raise the possibility that elevated intrinsic progranulin expression may contribute to the relative resistance of ExNeu *RNF220*− to FTD-associated neurodegeneration.

Conversely, no differentially expressed genes were identified in AD compared to control neuronal populations.

### 4. TDP-43-associated *STMN2* cryptic splicing selectively affects vulnerable excitatory neurons

Because FTD-*GRN* is characterized by TDP-43 proteinopathy, we next investigated whether thalamic neuronal populations exhibit differential susceptibility to TDP-43-associated splicing dysfunction (Figure 4A). We quantified cryptic exon inclusion within *STMN2*, a well-established marker of TDP-43 nuclear loss-of-function, across thalamic neuronal populations (Figure 4B, Supplementary Figure 4E). Despite technical challenges arising from the sequencing chemistry, Gittings et al. developed a robust bioinformatics approach to quantify splice junctions from 3’-end single-nucleus sequencing data (Gittings et al., 2023). Importantly, the *STMN2* cryptic exon is readily detectable using 3′-end sequencing because its inclusion introduces a premature stop codon, producing a truncated transcript that is captured by this chemistry. The same method was used in the current study to extract *STMN2* splice junctions and quantify reads to detect cryptic exons.

ExNeu *RNF220*+ neurons exhibited robust accumulation of *STMN2* cryptic exon transcripts selectively in FTD samples, indicating pronounced TDP-43-associated splicing dysfunction within this vulnerable excitatory neuronal population (Figure 4C, Supplementary Figure 4A). In contrast, *STMN2* cryptic exon transcripts were virtually absent in resilient ExNeu *RNF220*− neurons and across all AD samples, including cases with LATE stage 3 TDP-43 pathology (Figure 4D; Supplementary Figure 4B; **Supplementary Table 1**), supporting the specificity of this pathological signature within vulnerable FTD neuronal populations. Although low levels of cryptic exon inclusion were occasionally detected in inhibitory neuronal populations in FTD (Supplementary Figure 4C,D), limited junction coverage, and number of cells, precluded robust quantification (Figure 4E,F). Consistent with these observations, TDP-43 pathology was not detected in the rare remaining GAD65+ inhibitory neurons in FTD-*GRN* tissue, but only in excitatory neurons (Figure 4G). Interestingly, in control cases, while the expression of *TARDBP* (encoding the TDP-43 protein) was similar across all four neuronal populations, *STMN2* expression was higher in the resilient *RNF220*− neuronal population (Supplementary Figure 2F), a pattern mirroring *GRN* expression that could be protective against TDP-43 loss-of-function. Furthermore, elevated *STMN2* expression was consistently maintained in ExNeu *RNF220*− across AD, FTD-*GRN* and sFTD-TDP cases. In contrast, *TARDBP* expression was slightly upregulated in ExNeu *RNF220*−, specifically in FTD-*GRN* and sFTD-TDP (Supplementary Figure 2G).

Finally, to resolve the functional subtype-specific distribution of TDP-43 splicing pathology, we mapped individual nuclei harboring *STMN2* cryptic exon transcripts directly onto the thalamic neuronal UMAP (Figure 4H). Within the vulnerable ExNeu *RNF220*+ population, *STMN2* cryptic exon inclusion was selectively enriched within *CALB2*+ matrix excitatory neurons, whereas *PVALB*+ core excitatory neurons were largely spared (Figure 4H,I). These findings demonstrate that TDP-43-associated splicing pathology specifically targets higher-order matrix excitatory circuits within the FTD thalamus.

### 5. Selective disruption of thalamic neuronal networks in FTD

To determine how selective neuronal vulnerability alters thalamic circuit organization, we next inferred cell-cell communication networks across neuronal populations. FTD samples demonstrated marked disruption of both excitatory and inhibitory signaling pathways within the thalamus (Figure 5A,B). Both inhibitory neuronal populations demonstrated substantial reductions in GABAergic signaling interactions, consistent with widespread disruption of inhibitory thalamic circuitry (Figure 5C). Profiling of GABA-A pathway machinery across cell types revealed the specific molecular substrates underlying this circuit breakdown. In control individuals, inhibitory neuron populations (InNeu *SOX14*+ and InNeu *SOX14*−) expressed high baseline levels of GABA synthesis enzymes (*GAD1*, *GAD2*) as well as vesicular (*SLC32A1*) and plasma membrane (*SLC6A1*, *SLC6A11*) GABA transporters. By contrast, In FTD, InNeu *SOX14*− InNeu exhibited marked reductions in both synthesis enzymes (*GAD1*, *GAD2*) and transporter expression (Figure 5E).

Similarly, vulnerable ExNeu *RNF220*+ neurons exhibited a pronounced reduction in glutamatergic signaling interactions with neighboring cell populations, indicating impaired excitatory network communication in disease. In contrast, glutamatergic signaling originating from resilient ExNeu *RNF220*− neurons remained comparatively preserved across disease conditions (Figure 5D). Profiling of glutamate receptors, synthesis enzymes, and transporters revealed distinct molecular patterns between excitatory subtypes. ExNeu *RNF220*+ neurons displayed downregulation across multiple classes of glutamatergic machinery in FTD, including key NMDA receptor subunits (*GRIN1*, *GRIN2A*, *GRIN2B*), kainate receptor subunits (*GRIK3*, *GRIK4*), the AMPA receptor subunit *GRIA3*, the metabotropic receptor *GRM5*, the glutaminase enzyme *GLS*, and the glutamate transporter *SLC1A1* (Figure 5F). This coordinated transcriptomic decline indicates profound functional degradation of the glutamatergic apparatus within vulnerable excitatory circuits. In contrast, ExNeu *RNF220*− neurons maintained a comparatively stable expression profile across disease states, with only reductions observed in select NMDA subunits (*GRIN2A*, *GRIN2B*, *GRIN2D*), the metabotropic receptor *GRM1*, and the glutaminase gene *GLS2* (Figure 5F). Crucially, primary signaling components such as the vesicular glutamate transporter *SLC17A6* remained robustly expressed, reflecting relative preservation of core transmission infrastructure in this resilient population.

Together, these findings indicate that selective degeneration of vulnerable neuronal populations is accompanied by functional remodeling of thalamic and potentially extra-thalamic communication networks. Preservation of signaling output from ExNeu *RNF220*− neurons further supports the notion that this population maintains relative functional resilience despite widespread neurodegeneration.

### 6. Intrinsic molecular programs associated with neuronal resilience

Given the relative preservation of ExNeu *RNF220*− neurons across transcriptional, splicing, and communication analyses, we next sought to identify intrinsic molecular programs associated with neuronal resilience. Direct comparison revealed that resilient neurons are intrinsically equipped with a robust structural, metabolic, and proteostatic framework (Figure 6A–C, **Supplementary Table 5**). Pathway enrichment showed a pronounced baseline upregulation of axon maintenance and structural integrity programs, characterized by the high expression of neurofilaments (*NEFL*, *NEFM*), tubulins (*TUBB3*, *TUBA1B*), and microtubule-associated proteins (*MAP1B*). This rigid cytoskeletal backbone is supported by an active bioenergetic engine (*GAPDH*, *ATP1B1*, *ATP1A3*) and a deep reservoir of molecular chaperones and protein-clearing enzymes (*HSP90AA1*, *HSP90AB1*, *CLU*, *PSAP*, *SERINC1*, *UCHL1*). This heavily fortified homeostatic infrastructure (usually lost in neurodegeneration) drives a strong baseline enrichment for the “pathways of neurodegeneration - multiple diseases” term (hsa05022), providing these cells with the defensive reserve necessary to resist subsequent disease-associated proteotoxicity.

In contrast, vulnerable ExNeu *RNF220*+ neurons exhibit a baseline focus tailored toward highly specialized neuromodulation and high-amplitude signaling. The *RNF220*+ cluster is significantly enriched for the cholinergic synapse pathway, driven by the muscarinic receptor CHRM3, the intracellular calcium release channel ITPR2, and the voltage-gated potassium channel KCNQ3, alongside synaptic scaffolding and guidance factors (*NTNG1*, *FGF13*, *PPFIBP1*). While this configuration supports sophisticated signaling under healthy conditions, it leaves *RNF220*+ neurons baseline-configured for intensive ion-channel gating, high metabolic demands, and constant intracellular calcium flux. Together, these data suggest a distinct molecular divergence between the structural and proteostatic baseline of *RNF220*− cells and the signaling-centric organization of the *RNF220*+ population.

### 7. Vascular, immune, and glial alterations in FTD thalamus

Given the expansion of vascular-associated and immune populations observed in FTD, we next examined disease-associated transcriptional changes across non-neuronal cell types, which were isolated and visualized in individual UMAPs (Supplementary Figures 5 and 6). Blood vessel-associated cells and CD8+ lymphocytes were significantly increased in abundance relative to controls, but showed relatively limited transcriptional remodeling, suggesting that their expansion may primarily reflect altered blood-brain barrier permeability or immune recruitment rather than extensive intrinsic changes (Figure 7A–C, Supplementary Figure 5E).

Among glial populations, astrocytes exhibited the most pronounced transcriptional remodeling, characterized by reduced expression of genes involved in neurotransmitter homeostasis and neuronglia communication, including *SLC1A2, GRIA2,* and *CACNG2*, together with increased expression of extracellular matrix remodeling and vascular-associated genes such as *SPARC, CCN2*, and *COL8A1* (Figure 7D,E, **Supplementary Tables 3 and 4**). Oligodendrocytes exhibited a more modest response marked by reduced expression of genes involved in lipid biosynthesis (*FADS3*, *PLCL2*) alongside those governing membrane biogenesis, cell projection organization, and the regulation of oligodendrocyte differentiation (*OPALIN*, *TMEM98*, *ZNF365*). Conversely, transcriptional remodeling was defined by the increased expression of genes driving cell-cell junctions and axo-glial adhesion (*PCDH20*, *PGM5*), active remyelination (*RXRG*), and lipid raft dynamics associated with membrane biogenesis and cell projection organization (*CAV1*, *CAV2*) (Figure 7F,G, **Supplementary Tables 3 and 4**). By comparison, microglia exhibited more modest transcriptional shifts, marked by the upregulation of lipid-associated *APOE* and lysosomal *GPNMB* amid increases in positive chemotaxis, extracellular matrix, and intracellular signaling cassettes, with concurrent negative regulation of cellular component organization (Figure 7H,I, **Supplementary Tables 3 and 4**).

Together, these findings indicate that non-neuronal alterations in the FTD thalamus are dominated by astrocytic remodeling, with more modest oligodendrocyte and microglial responses.

By contrast, transcriptional remodeling in the AD thalamus was markedly limited relative to controls. Glial cell types collectively exhibited only ten DEGs: four up-regulated (*DNAJB9, UQCC3, SCD, ARMCX3*) and five down-regulated (*CCDC194, ZSCAN31, HOPX, RIN3, MSTO1*) in astrocytes, and a single down-regulated gene in BVCs (*RALGPS1*).

Overall, our integrated analysis reveals that the human thalamus exhibits highly selective neuronal vulnerability in FTD characterized by degeneration of discrete inhibitory and excitatory neuronal populations, disruption of thalamic communication networks, as well as glial and vascular remodeling. In parallel, we identify a resilient excitatory neuronal population defined by elevated *GRN* expression, preserved neuronal signaling, resistance to TDP-43-associated *STMN2* cryptic splicing, and enrichment of cytoskeletal maintenance programs, providing a potential molecular framework for neuronal resilience in human neurodegeneration.

## Discussion:

Using integrated single-nucleus transcriptomics across three independent human cohorts, we generated a cellular atlas of the human thalamus in neurodegeneration, and identified highly selective neuronal vulnerability in frontotemporal dementia. While Alzheimer’s disease samples exhibited relatively preserved thalamic cellular composition, FTD was characterized by highly selective degeneration of discrete inhibitory and excitatory neuronal populations together with parallel vascular, immune, and glial alterations. These findings establish the antero-median thalamus as a selectively vulnerable subcortical region in FTD and provide a cellular framework linking thalamic degeneration to circuit dysfunction.

A central finding of this study is that inhibitory neuronal degeneration is a robust, conserved feature of the FTD thalamic landscape. While prior work noted general inhibitory cell loss within a single FTD-*GRN* cohort (Marsan et al., 2023), our cross-cohort single-cell analysis resolves the molecular identity and reproducibility of this phenotype by demonstrating the concurrent depletion of two distinct inhibitory populations, *SOX14*+ and *SOX14*−, across independent global cohorts. This selective loss was not mirrored across other thalamic neuronal populations. Ontologically, *SOX14*+ inhibitory neurons map to developmentally specialized midbrain-derived circuits, while *SOX14*− inhibitory neurons, which encompass *PVALB*+, *SST*+ and *LAMP5*+ neurons, align with forebrain lineages (Bakken et al., 2021; Jager et al., 2021). Because both groups are depleted despite these distinct developmental trajectories, their vulnerability is unlikely to be driven by lineage-specific traits alone. Instead, our findings point to a multi-layered mechanism: a baseline vulnerability inherent to inhibitory neurons in FTD, compounded by strict thalamic circuit-specific demands. This subcortical pan-inhibitory deficit conceptually aligns with reported impairment of cortical *PVALB*+ interneurons in FTD frontal cortex (Marsan et al., 2023), suggesting that interneuron susceptibility may be a widespread feature across multiple brain regions. Within the thalamus, the severe depletion of both inhibitory neuron populations suggests that unique circuit architecture or local metabolic pressures may aggravate this systemic vulnerability. This circuit-level strain is tentatively supported by the focal downregulation of *KCNAB1*, a potassium voltage-gated channel subunit gene essential for modulating intrinsic excitability, within the remaining *SOX14*− population.

In addition to inhibitory neuronal degeneration, our analyses identified divergent responses among excitatory neuronal populations. Vulnerable ExNeu *RNF220*+ neurons exhibited extensive transcriptional dysregulation, impaired glutamatergic communication, and robust accumulation of *STMN2* cryptic exon transcripts, indicating pronounced susceptibility to TDP-43-associated proteinopathy. Notably, *ExNeu RNF220*+ neurons exhibited a coordinated upregulation of cholesterol biosynthetic, sensing, and trafficking genes, suggesting that lipid dysregulation is a major feature of vulnerable neuronal state. These findings are particularly intriguing given the established role of progranulin in lysosomal biology and lipid homeostasis (Evers et al., 2017; Filiano et al., 2013; Khrouf et al., 2023; Ward et al., 2017). Our data extend these observations to selectively vulnerable thalamic neurons raising the possibility that altered cholesterol metabolism represents an early component of the cell-intrinsic neuronal stress response in FTD-*GRN*. This metabolic disruption coincided with extensive structural remodeling, characterized by internal actin dynamics, cell-matrix detachment, and synaptic stress. This progressive dismantling likely destabilizes synapses and primes vulnerable neurons for microglial-mediated elimination.

By contrast, ExNeu *RNF220*− neurons remained comparatively preserved and retained their baseline glutamatergic signaling output despite exposure to the same disease environment. This resilient population exhibited elevated *GRN* expression in control tissue, and a striking protection from *STMN2* cryptic splicing pathology. Given that the loss of nuclear TDP-43 function typically results in *STMN2* cryptic exon inclusion, the absence of these transcripts in patients suggests that *STMN2* cryptic splicing is a selective marker of impending functional collapse rather than a universal feature of the diseased thalamus. These control tissue profiles suggest that resilience in the FTD thalamus is an active biological state. Resilient neurons possess a superior baseline infrastructure defined by fortified cytoskeletal, bioenergetic, and proteostatic machinery, coupled with higher intrinsic expression of progranulin and stathmin-2. This contrasts sharply with the vulnerable cluster, whose baseline prioritization of high-demand synaptic signaling creates an intrinsic metabolic liability under subsequent disease stress.

Intriguingly, spatial transcriptomics localized control ExNeu *RNF220*+ neurons to the medial thalamic nuclei. In our single-cell dataset, TDP-43 pathology related *STMN2* CEs were enriched in ExNeu *RNF220*+ cells, particularly within CALB2+ subset of cells, consistent with midline/intralaminar nuclei. Because these vulnerable *RNF220*+ projection neurons and co-localized *SOX14*+ inhibitory populations participate in conserved associative loops (Sherman & Usrey, 2024), their disruption may undermine the feed-forward modulation and inhibitory gating essential for cortical network stability. Specifically, the resulting loss of shunting inhibition suggests a cellular basis for the macro-scale thalamocortical hyperconnectivity observed in presymptomatic *FTD-GRN* carriers (Lee et al., 2019). Ultimately, this selective collapse indicates that FTD may represent a progressive breakdown of thalamocortical network integration rather than isolated cortical dysfunction. Because standard rodent models lack part of these human-specific circuit architectures and inhibitory neuron subpopulations (Kapustina et al., 2024; Marsan et al., 2023; Phillips et al., 2019; Roy et al., 2022), our findings highlight the critical need for human-relevant translational platforms, such as patterned thalamic organoids, to model these disease mechanisms.

Although most of our analyses focused on FTD-*GRN*, the inclusion of three sporadic FTD-TDP cases provided preliminary evidence that key features of thalamic vulnerability may extend beyond progranulin-associated disease. While the limited number of sporadic cases precluded definitive statistical comparisons, vulnerable neuronal populations exhibited similar patterns of degeneration and molecular dysregulation, suggesting that at least some aspects of thalamic vulnerability may represent convergent consequences of TDP-43 proteinopathy. Larger cohorts of sporadic FTD-TDP cases will be required to determine the extent to which the vulnerability and resilience programs identified here generalize across FTD subtypes.

This neuronal degeneration did not occur in isolation but was accompanied by distinct non-neuronal shifts. While the expansion of vascular cells and lymphocytes with limited transcriptional remodeling likely reflects secondary blood-brain barrier permeability or immune recruitment, astrocytes, oligodendrocytes, and microglia demonstrated broad, systemic transcriptional responses consistent with reactive gliosis (Gerrits et al., 2022; Marsan et al., 2023). In particular, astrocytes shifted away from homeostatic neurotransmitter support toward vascular and matrix remodeling, while oligodendrocytes suppressed mature myelination in favor of reactive axo-glial adhesion dynamics. Coupled with microglial lipid and lysosomal clearance responses, this integrated landscape indicates that macroglial remodeling actively mirrors the structural and metabolic instability observed in the vulnerable neuronal compartments.

This study has several limitations. Although integration across multiple independent cohorts substantially improves robustness and reproducibility, the rarity of human FTD tissue limits sample size for certain subgroup analyses. In addition, transcriptomic profiling alone cannot fully resolve causal relationships between progranulin deficiency, TDP-43 pathology, and neuronal degeneration. Furthermore, the relatively low recovery of specific cell types, such as the inhibitory neuronal populations, may impact the statistical power to detect differentially expressed genes (DEGs) and characterize low-abundance splicing events like cryptic exon inclusion. Future functional studies in human-derived systems will therefore be required to determine whether the resilience-associated programs identified in ExNeu *RNF220*− neurons directly confer neuroprotection.

Overall, our findings define a cellular and molecular framework for selective thalamic vulnerability in human FTD. By identifying neuronal populations that are either highly susceptible or relatively resistant to degeneration, this work provides insight into the mechanisms governing selective neurodegeneration and highlights endogenous resilience pathways that may represent potential therapeutic targets in FTD and related TDP-43 proteinopathies. More broadly, our study demonstrates the power of multi-cohort single-cell integration to resolve reproducible disease-associated cellular states in rare human neurodegenerative disorders.

## Methods:

### snRNA-Seq datasets

Data were obtained from two published datasets (AD: Mathys et al. 2024 syn52383413; and FTD: Marsan et al. 2023 PRJNA644744) and a newly generated cohort of FTD-*GRN* and sporadic FTD-TDP cases reported here in Trujillo et al. Metadata including donor age, sex, brain region, postmortem interval, and neuropathological diagnosis were curated to ensure consistency across datasets (Supplementary Table 1).

In brief, cases from Mathys et al. 2024 clinical and neuropathological diagnosis were obtained from Mathys Suppl Table1 and ROSMAP basic metadata as follow: Control: Suppl Table 1 > pathAD = non-AD and basic > niareagansc: No AD = 4 or low = 3; AD: Suppl Table 1 > pathAD = AD and basic > niareagansc: intermediate = 2 or high = 1.

### Sample preparation for 10X single nucleus RNA sequencing (snRNA-Seq)

Frozen postmortem brain tissue from FTD-*GRN*, sporadic FTD-TDP, and matched controls were obtained from Paris PBI “NeuroCEB” Brain Bank. Nuclei were isolated using a detergent-based lysis protocol, filtered, and sorted to remove debris. Single nuclei were loaded onto the 10X Chromium platform following the manufacturer’s protocol, and cDNA libraries were generated and sequenced on an Illumina platform to a target depth of 50,000 reads per nucleus.

### snRNA-Seq harmonization, doublet exclusion and cell type annotation

FASTQs were downloaded from Synapse and NCBI for the published AD and FTD datasets, respectively. All data was mapped to the reference genome (GRCh38) and reads were counted using cellranger (v8.0.1)(Zheng et al., 2017). Ambient RNA was then removed using cellbender (v0.3.2)(Fleming et al., 2023). For each individual, standard quality control (QC) metrics were used to remove low quality nuclei (mitochondrial RNA > 20%, ribosomal RNA > 20%, UMI count < 200, and Gene count < 200). Individuals (FLTD-GRN1, D19-12386) with less than 800 cells remaining after QC filtering were removed. Filtered read count matrices were then log-normalized and concatenated for each study separately. All individuals for each study were then integrated based on SampleID using mnc correct (snapatac2)(Zhang et al., 2024). Doublets were identified and manually removed through an iterative process involving unsupervised Leiden clustering and dimension reduction (UMAP), followed by examination of feature plots using marker genes using Scanpy (Wolf et al., 2018). Individuals (Control10, D19-12393) were with less than 800 cells after doublet clusters were removed. The data was then integrated across all datasets, again, based on SampleID using mnc correct and further manual removal of doublets, as above, was performed. Once the final, integrated UMAP was complete, cell types were manually annotated based on canonical marker genes (Supplementary Figure 1). Individual 3549-T formed its own clusters and was therefore removed. Inhibitory neuron subtypes, including *SOX14*− and *SOX14*+, and excitatory neuron subtypes, including *RNF220*+ and *RNF220*− were identified using normalized gene expression of *SOX14* and *RNF220*.

### Cell type proportion analysis

To account for differences between cohorts and Disease status in cell type proportions, Propeller (Phipson et al., 2022) was used to analyze differential cell type proportions. First, transformed proportions were calculated using getTransformedProps arcsin square root transformation based on annotated cell type clusters and SampleIDs. Then, using the transformed proportions, limma-voom (Ritchie et al., 2015) was used to include ‘Cohort’ as a blocking variable and contrasts (FTD vs. Control and AD vs. Control) were examined with the following model [Transformed proportion ~ Disease status + (1 ∣ study)]. To further investigate neuron subcluster differential cell type proportions, the above model was applied to the entire dataset, but this time including more granular cell types within the neuron cluster (InNeu *SOX14*+, InNeu *SOX14*−, ExNeu *RNF220*+, and ExNeu *RNF220*−).

### Differential expression, gene ontology

To identify disease-associated differentially expressed genes (DEGs), samples were stratified by cohort of origin and the two FTD cohorts (Biogen and Marsan) were pooled to maximize statistical power prior to pseudobulk aggregation. Pseudobulk differential expression was performed separately for each cell type using DESeq2(Love et al., 2014). For the FTD analysis, unexplained variation was removed using RUVSeq(Risso et al., 2014), with the optimal number of factors of unexplained variation (k) determined independently for each cell type via variance partitioning (k = 2 for neuronal subtypes and microglia/astrocytes; k = 5 for oligodendrocytes). Collinearity among covariates was assessed using variance inflation factors (VIF), with any covariate exceeding a threshold of VIF > 5 excluded from the final model.All models included disease status (FTD vs. Control), sex, age at death, and k RUVSeq-derived factors of unexplained variation (W_1_–W*_k_*) as follow: Neuronal subtypes: [Read counts ~ Disease status + Sex + AgeDeath + Cohort + W_1_…W*_k_*], Microglia, Astrocytes, and Oligodendrocytes: [Read counts ~ Disease status + Sex + AgeDeath + W_1_…W*_k_*]. DEGs were then corrected for multiple comparisons using Benjamini-Hochberg (BH) FDR correction and final log2 fold change estimates were shrunk toward zero using lfcShrink(). To assess functional enrichment of DEGs, genes meeting a significance threshold of adjusted p < 0.05 and ∣log_2_FC∣ > 0.5 were submitted to Metascape(Zhou et al., 2019) for pathway and gene ontology analysis. When possible (for Oligodendrocyte, Astrocyte and ExNeu *RNF220*+, but not Microglia clusters), up- and down-regulated protein coding DEGs were submitted as two separated lists using metascape custom analysis. Only Pathway>GO Biological Processes, KEGG Pathway and Structural Complex>GO Cellular Components were selected for enrichment analysis.

To further explore differences in expression between resilient and vulnerable excitatory neuron subtypes, the atlas was subset to include only ExNeu *RNF220*+ and ExNeu *RNF220*− cells. Then a one-versus-rest Wilcoxon test was run with sc.tl.rank_genes_groups() using scanpy (Wolf et al., 2018) and a BH FDR correction was implemented to get p-adj. Top 100 significantly up-regulated protein coding DEGs (padj<0.05, LogFC>0.5, excluding uncharacterized genes, and ordered by z-score) were run through metascape custom analysis for pathway and gene ontology analysis. Only Pathway>GO Biological Processes, KEGG Pathway and Structural Complex>GO Cellular Components were selected for enrichment analysis.

As all AD samples were derived from a single cohort (Mathys et al.), RUVSeq correction was not applied, and study was excluded as a covariate. For each cell type, the model included disease status (AD vs. Control), sex, age at death, and post-mortem interval (PMI) as covariates. Differentially expressed genes were identified using DESeq2, with multiple comparisons corrected using the Benjamini-Hochberg (BH) procedure and log2 fold change estimates stabilized using lfcShrink().

### *STMN2* cryptic exon detection

Splice junctions were extracted and quantified as described by Gittings et al. (Gittings et al., 2023). Neuron-annotated post-QC single-cell BAM files were used to filter reads mapped to *STMN2* using samtools view (v1.19.2)(Li et al., 2009). Splice junctions were then extracted in parallel using regtools junctions extract (v1.0.0)(Cotto et al., 2023), and junction counts were summarized using LeafCutter’s leafcutter_cluster_regtools.py(Li et al., 2018) in Python (v3.9.15). This yielded a junction-by-barcode matrix containing four *STMN2* junctions across 46,253 barcodes, after further removing rarely used junctions by LeafCutter. These junctions were chr8:79611214:79616822, chr8:79611214:79636802, chr8:79611433:79636802, and chr8:79611791:79636802, following the <chr>:<start>:<end> format.

After junctions were annotated based on overlapping genomic loci using the same GTF annotation file used for read mapping, splice types were classified according to exon boundary matching junctions whose start and/or end coordinates matched annotated exon boundaries as non-cryptic, whereas junctions those not matching the boundaries were classified as cryptic. Exons were further categorized by whether they generated cryptic or non-cryptic structures, alongside a subclassification as annotated or novel based on boundary concordance. The current analysis did not detect any non-cryptic-novel exons for *STMN2*.

For each sample, relative cryptic splicing was assessed within each cell subtype and disease condition. To quantify this, *STMN2* junction counts were aggregated by exon type on a per-cell basis within each cell subtype and disease condition. Samples with zero total junction counts were excluded prior to log-transformation. Counts were transformed to a natural logarithmic scale with a pseudocount of 1. The ratio of TDP43-related *STMN2* cryptic junction counts to total detected junction counts was then calculated after adding a pseudocount of 1/10 of the minimum non-zero value within each cell subtype and disease condition. Statistical significance between the disease and control groups was analyzed using a two-tailed Wilcoxon rank-sum test with the wilcox.test function in R v4.3.3.

For Sashimi plots, per-barcode BAM files were aggregated into per-subset BAM files, representing cell subtype within disease condition, using samtools cat. These files were subsequently sorted and indexed using samtools sort and samtools index. Sashimi plots were generated using the Integrative Genome Browser (IGV, v2.16.0)(Robinson et al., 2011) with the minimum displayed junction count set to 4. Reference tracks were generated by extracting *STMN2* exons from the GTF file in R using the rtracklayer package (v1.66.0)(Lawrence et al., 2009) and exporting both exon and junction coordinates as BED files. Line colors and styles were edited using Adobe Illustrator. The complete codebase, file processing pipeline, and plotting details are available in the project repository (https://github.com/NIH-CARD/CARD_MPU/tree/main/Thalamus_atlas).

### Cell-cell interaction analyses

To investigate the overall differences in cell-cell communication between FTD and control cases from the same cohorts, read count data was leveraged to find meaningful ligand-receptor (L-R) interactions using Cellchat (Jin et al., 2025). First, samples were split by condition (FTD and control) and run separately to obtain significant L-R interactions (interaction number) and their probability scores (interaction strength). Then, to find changes in cell-cell communication between Disease status (FTD and control), we merged each separate dataset using mergeCellChat() and computed differential interaction strength (Probability_FTD_-Probability_Control_) and differential interaction number (interaction number_FTD_-interaction number_Control_). Differential interaction strength and differential interaction number were examined across all cell types, followed by neuronal-specific pathway-level comparisons of communication strength between FTD and control. To ensure visual comparability across conditions, edge weights in circle plots were normalized relative to the maximum weight across all compared conditions.

### Spatial transcriptomics

We obtained the preprocessed 10x Genomics Visium dataset previously generated and published by Schulmann et al. (Schulmann et al., 2024) (Schulmann et al. *under revision*). Briefly, 14μm-thick fresh frozen coronal sections from the thalamus of a 33-year-old white male with no history of neuropsychiatric disorders were processed via hematoxylin and eosin (H&E) staining, followed by sequencing on an Illumina NextSeq2000 system. Sequencing reads were mapped to the human probe set v2.0 using 10x Genomics SpaceRanger v2.1.1. We performed secondary downstream analysis using the pre-filtered Seurat object, from which low-quality spots were removed as described by Schulmann et al. Marker gene expression was visualized using the SpatialFeaturePlot function from the Seurat package (v5.3.0)(Hao et al., 2024) in R v4.3.3.

### Immunostains, microscopy imaging and quantification

Formalin-fixed paraffin-embedded sections (10 μm thick) were acquired from the Neuro CEB brain bank. Sections were baked for 1 h at 60 °C, deparaffinized with two consecutive 10 min immersions in xylene, rehydrated through graded ethanol washes (100% twice, 96% twice, 70% once for 3 min each), and rinsed in distilled water for 5 min. Then, sections underwent heat-induced antigen retrieval in a temperature-controlled chamber using a citrate-based buffer, pH 6.0 (Vector Laboratories, cat# H-3300-250), for 30 min at 95 °C. Following antigen retrieval, slides were cooled for 20 min at room temperature in the buffer, then permeabilized in TBS containing 0.1% Triton X-100 for 10 min, and blocked in TBS containing 3% BSA for 1 h at room temperature. Primary antibodies including, chicken anti-MAP2 (Abcam, cat# ab5392, 1:250), rabbit anti-TDP-43 (N-terminal) (Proteintech, cat# 10782-2-AP, 1:250), and goat anti-GAD65 (R&D Systems, cat# AF2247, 1:40), were diluted in TBS containing 3% BSA and incubated overnight at room temperature in a humidified chamber. Sections were incubated for 1 h at room temperature with the following Alexa Fluor secondary antibodies diluted 1:500 in TBS containing 3% BSA: donkey anti-chicken 488 (Invitrogen, cat# A78948), donkey anti-rabbit 594 (Invitrogen, cat# A32754), and donkey anti-goat 647 (Invitrogen, cat#A32849). Sections were counterstained with DAPI (ThermoFisher Scientific, cat# 62248) diluted 1:500 in TBS for 15 min at room temperature. Subsequently, sections were treated with TrueBlack Lipofuscin Quencher (Biotium, cat# 23007) according to the manufacturer’s recommendation, mounted using ProLong Gold Antifade Mountant (ThermoFisher Scientific, cat# P36930), and coverslipped. Images were captured on an Olympus SLIDEVIEW VS200 slide scanner equipped with a Hamamatsu ORCA-Fusion Digital CMOS camera and a UPLXAPO 20x (NA 0.80) objective Virtual slide images were viewed on OlyVIA software (Evident Scientific).

## Supplementary Material

1

**Supplementary Table 1: Harmonized metadata for snRNA-seq cohorts.** Summary of donor characteristics for the three cohorts included in the integrated single nucleus RNA-seq analysis. Metadata includes SampleID, Brain region, Disease (i.e., FTD-*GRN*, sFTD-TDP, AD, and Control), Clinical diagnosis (ClinicalDx), Neuropathological diagnosis (NeuropathDx), TDP pathology (TDP_Path), Age at death (AgeDeath), Sex, Genetics, Postmortem interval (PMI - hours), RNA integrity number (Quality_RIN), Thal, Braak, CERAD, MMSE, DiseaseDuration, Cohort, status (i.e., Patient or Control), and Disease status (i.e., FTD, AD, Control), inclusion or exclusion in final atlas (snRNAseq_atlas). In the cohort column, ‘Trujillo’ represents the newly generated sFTD-TDP, FTD-*GRN* and control samples, while ‘Mathys.’ and ‘Marsan’ are previously published AD and FTD-*GRN* datasets. All data were harmonized to ensure consistent labeling and facilitate cross-cohort integration

**Supplementary Table 2: Cell type numbers per cohort included in the snRNAseq atlas.** Summary of the cell numbers for each top level brain cell type distributed across cohorts.

**Supplementary Table 3: Differentially expressed genes for all cell types.** Differentially expressed genes calculated for each cell type using DEseq2. All genes are included regardless of significance.

**Supplementary Table 4: Top 20 enriched pathways for ExNeu *RNF220*+, astrocytes, oligodendrocytes top DEGs and ExNeu subtypes top markers comparison.** Results for the Top 20 functional enrichment terms in the metascape analysis for ExNeu *RNF220*+, astrocytes, and oligodendrocytes comparing significantly up- (LogFC > 0.5) and down-regulated (LogFC < 0.5) DEGs with a significance cutoff of p-adj<0.05. Also included are the metascape results comparing the top 20 most significantly up-regulated (LogFC > 0, padj < 0.05, excluding uncharacterized genes, and ordered by z-score) genes of ExNeu subtypes (ExNeu *RNF220*+ and ExNeu *RNF220*−) as calculated using the Wilcoxon one versus rest test.

**Supplementary Table 5: Differentially expressed genes between excitatory neuron subtypes.** Differentially expressed genes calculated between ExNeu *RNF220*− and ExNeu *RNF220*+ using a Wilcoxon one versus rest test. All genes were included regardless of significance.

This is a list of supplementary files associated with this preprint. Click to download.

- SupplementaryTable1copy.xlsx
- SupplementaryTable1copy.xlsx
- SupplementaryTable1copy.xlsx
- SupplementaryTable1copy.xlsx
- SupplementaryTable1copy.xlsx
- SupplementaryTable1copy.xlsx
- SupplementaryTable2copy.xlsx
- SupplementaryTable2copy.xlsx
- SupplementaryTable2copy.xlsx
- SupplementaryTable2copy.xlsx
- SupplementaryTable2copy.xlsx
- SupplementaryTable2copy.xlsx
- SupplementaryTable4copy.xlsx
- SupplementaryTable4copy.xlsx
- SupplementaryTable4copy.xlsx
- SupplementaryTable4copy.xlsx
- SupplementaryTable4copy.xlsx
- SupplementaryTable4copy.xlsx
- SupplementaryTable3copy.xlsx
- SupplementaryTable3copy.xlsx
- SupplementaryTable3copy.xlsx
- SupplementaryTable3copy.xlsx
- SupplementaryTable3copy.xlsx
- SupplementaryTable3copy.xlsx
- SupplementaryTable5copy.xlsx
- SupplementaryTable5copy.xlsx
- SupplementaryTable5copy.xlsx
- SupplementaryTable5copy.xlsx
- SupplementaryTable5copy.xlsx
- SupplementaryTable5copy.xlsx

## Figures and Tables

**Figure 1:**
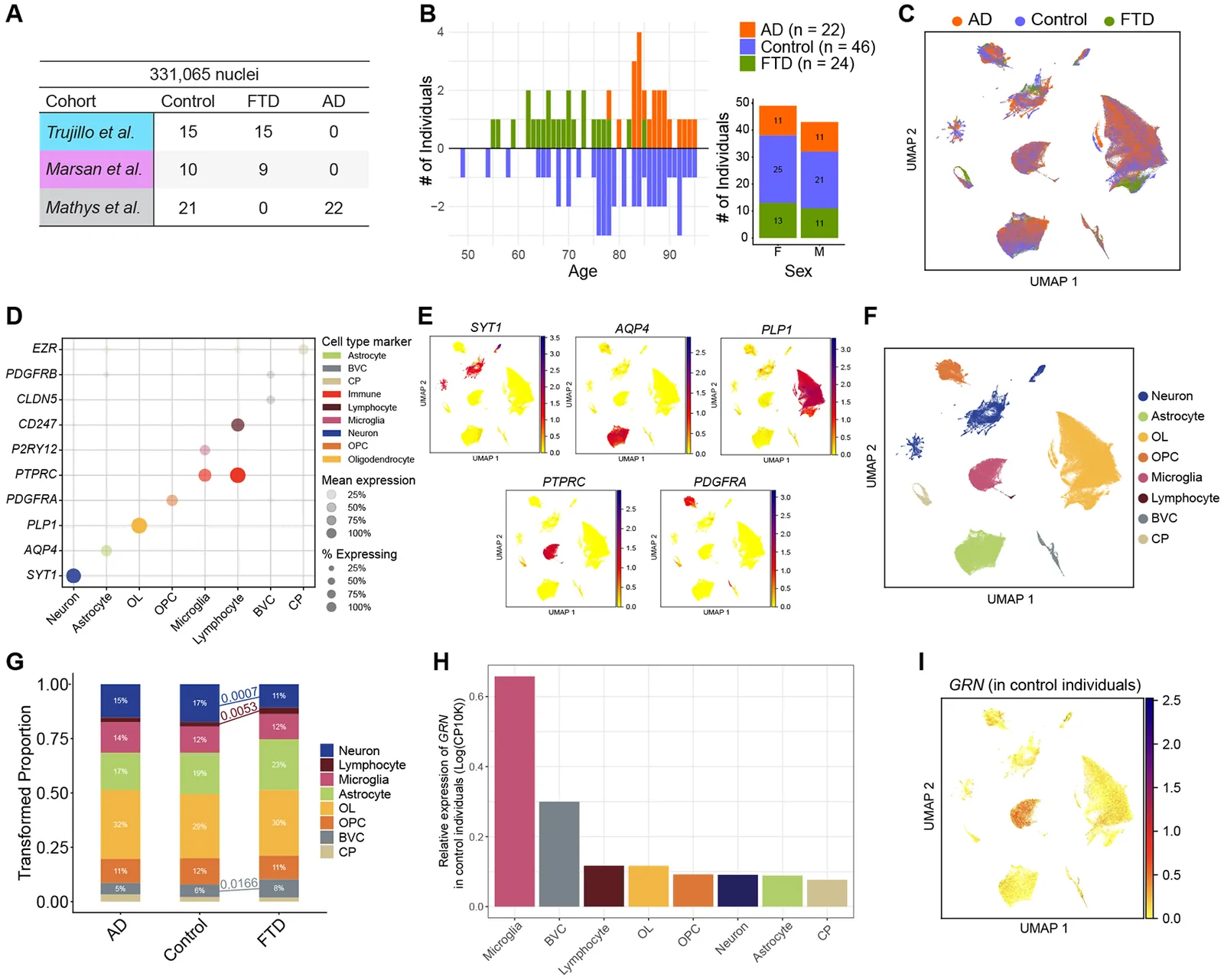
Single-nucleus transcriptomic atlas of the human thalamus in AD and FTD. (A) Schematic of the three cohorts used to build the thalamus snRNAseq atlas. (B) Age (left) and sex (right) distribution across the individuals included in the analyses. (C) UMAP projection of 331,065 nuclei colored by disease status. (D) Dot plot showing marker genes defining major brain cell types where dot size reflects the proportion of cells expressing each gene and color intensity reflects mean expression within each cell type. (E) UMAP feature plots showing expression levels of marker genes defining major brain cell types. Color intensity represents relative gene expression (counts per million). (F) UMAP projection colored by top level brain cell type markers. (G) Bar plot displaying transformed cell type proportions across control, AD, and FTD conditions, with significant adjusted p-values from differential cell type proportion analysis shown for neurons, lymphocytes, and BVC. Adjusted p-values were calculated by linear model using limma’s lmFit and moderated using eBayes. (H) Bar plot of the relative log-CP10K expression of *GRN* across all top level thalamic cell types in only control individuals. (I) UMAP feature plot showing the expression level of *GRN* in only control individuals. Color intensity represents relative gene expression (counts per million). Abbreviations: OL, oligodendrocyte; OPC, oligodendrocyte precursor cell; BVC, blood vessel cell; CP, choroid plexus.

**Figure 2:**
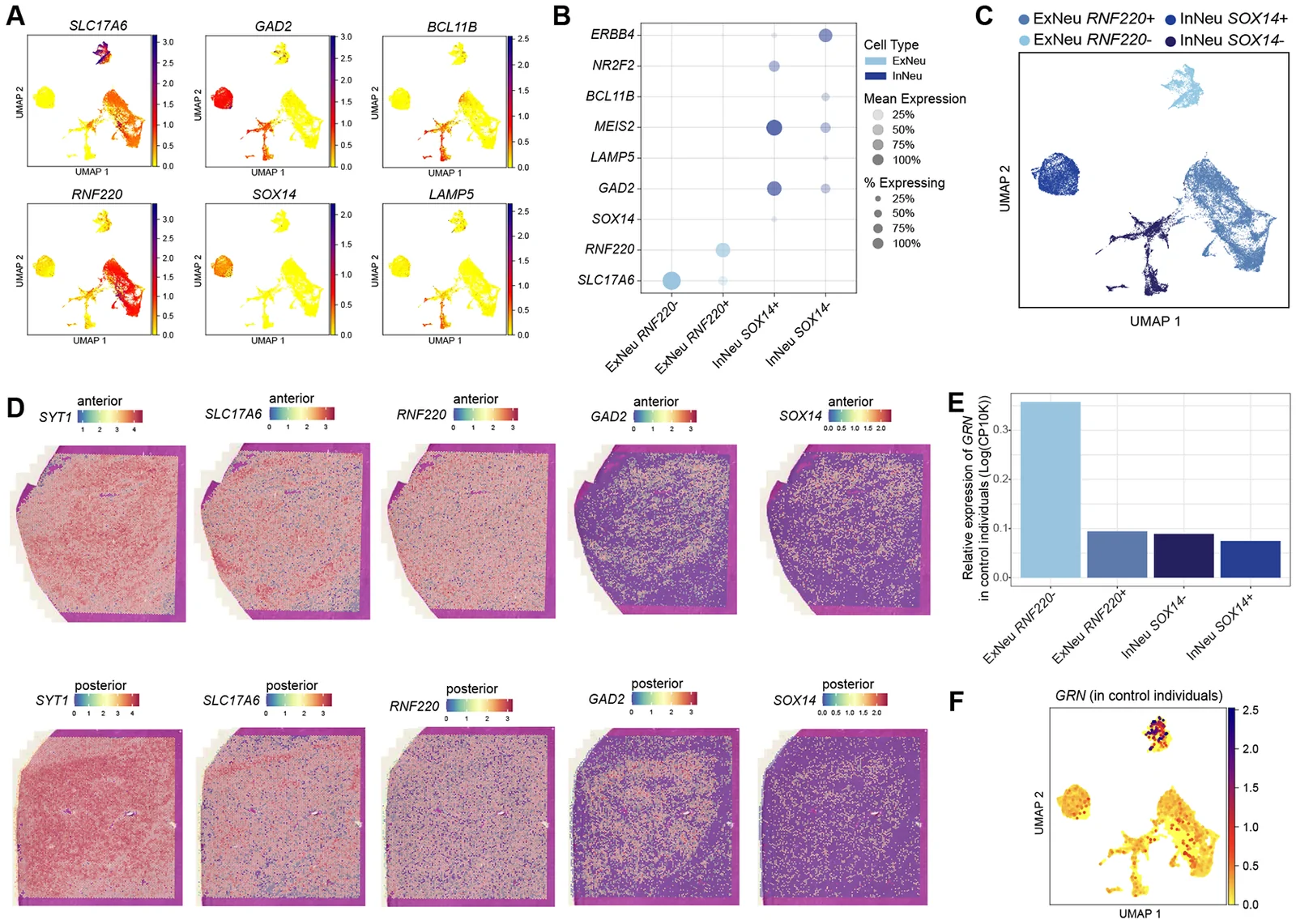
Molecular taxonomy of thalamic neurons. (A) UMAP feature plots showing expression levels of thalamic neuron subtypes marker genes. Color intensity represents relative gene expression (counts per million). (B) Dot plot showing marker gene expression profiles used to define these four subtypes, where dot size reflects the percentage of cells expressing each gene and color intensity reflects mean expression within each cell type. (C) UMAP visualization of two thalamic excitatory neuron (ExNeu) subtypes (ExNeu *RNF220*+, and ExNeu *RNF220*−) and two thalamic inhibitory neuron (InNeu) subtypes (InNeu *SOX14*+, InNeu *SOX14*−). (D) Spatially resolved expression of marker genes defining neuronal subtypes across two coronal sections from the adult human medial thalamus. (E) Bar plot of the relative log-CP10K expression of *GRN* across thalamic neuron subtypes in control individuals. (F) Feature plot of *GRN* expression in thalamic neuron subtypes from control individuals only. Color intensity represents relative gene expression (counts per million).

**Figure 3:**
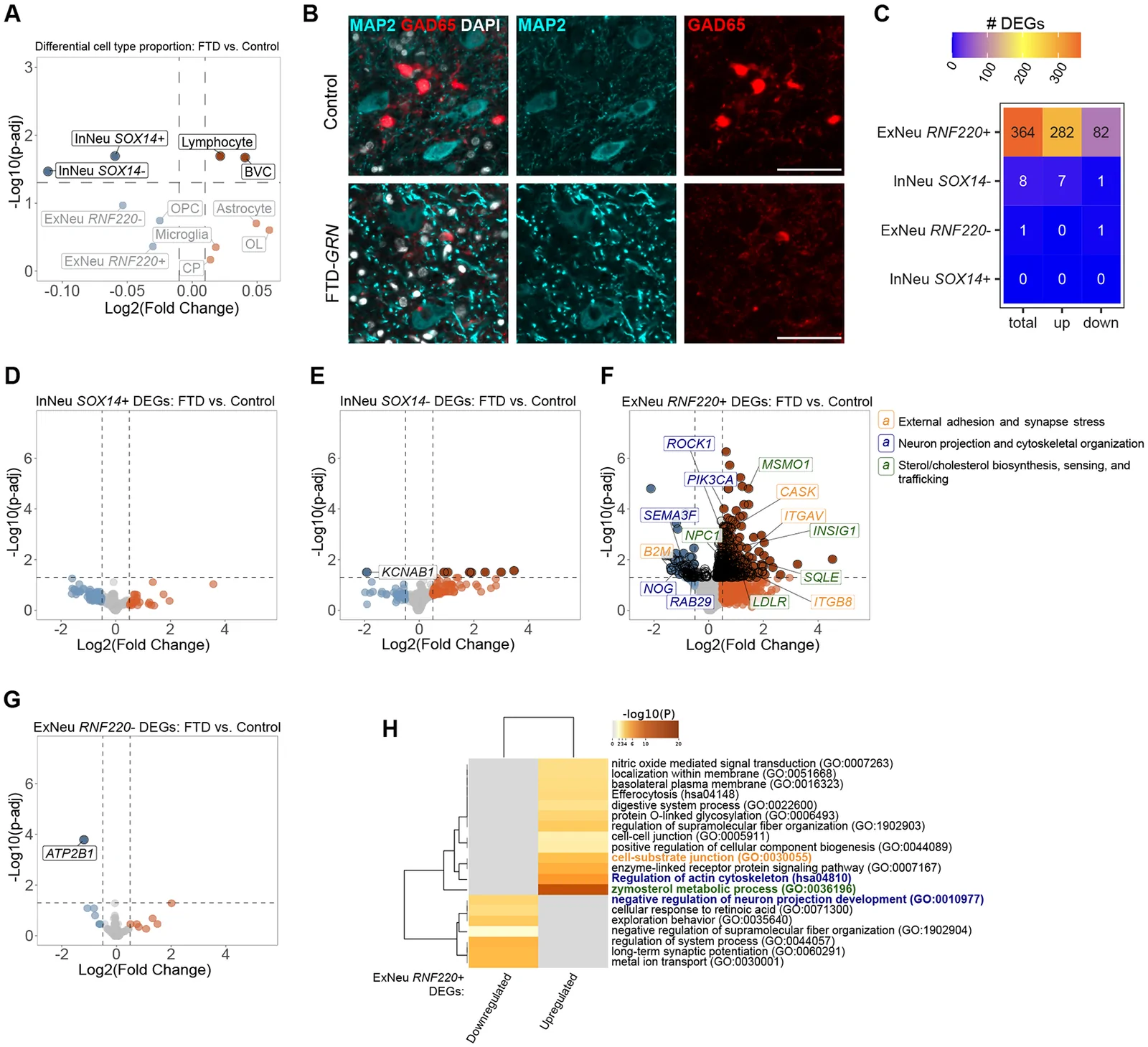
Selective neuronal vulnerability in FTD. (A) Volcano plot showing the differential cell type proportion across the brain cell types, including the four thalamic neuronal subtypes, where dotted lines represent the significance cutoff (p-adj<0.05 and ∣LogFC∣>0.01). (B) Immunofluorescence staining of neurons (MAP2), inhibitory neurons (GAD65) and nuclei (DAPI) in control and FTD-*GRN* thalamus. Scale bars = 50 μm. (C) Heatmap representing the numbers of total, up-regulated, and down-regulated significant differentially expressed genes (DEGs) across neuronal subtypes. DEGs were considered significant if p-adj<0.05 and ∣LogFC∣>0.05. (D-G) Volcano plots displaying expression changes and genes of interest identified between FTD and Control groups (positive LogFC = Up-regulated in FTD, negative LogFC = down-regulated in FTD, dotted lines represent significance threshold of p-adj<0.05 and ∣LogFC∣>0.5) within the InNeu *SOX14*+ (D), InNeu *SOX14*− (E), ExNeu *RNF220*+ (F), and ExNeu *RNF220*− (G) subtypes. (H) Metascape pathway enrichment analysis of differentially up- (LogFC<0.05 and p-adj<0.05) and down-regulated (LogFC<-0.05 and p-adj<0.05) genes within the ExNeu *RNF220*+ subtype. (I-K) relative Log(CP10K) expression in neuronal subtypes between AD, Control, and FTD for (D) *GRN*, (E) *STMN2*, and (F) *TARDBP*.

**Figure 4:**
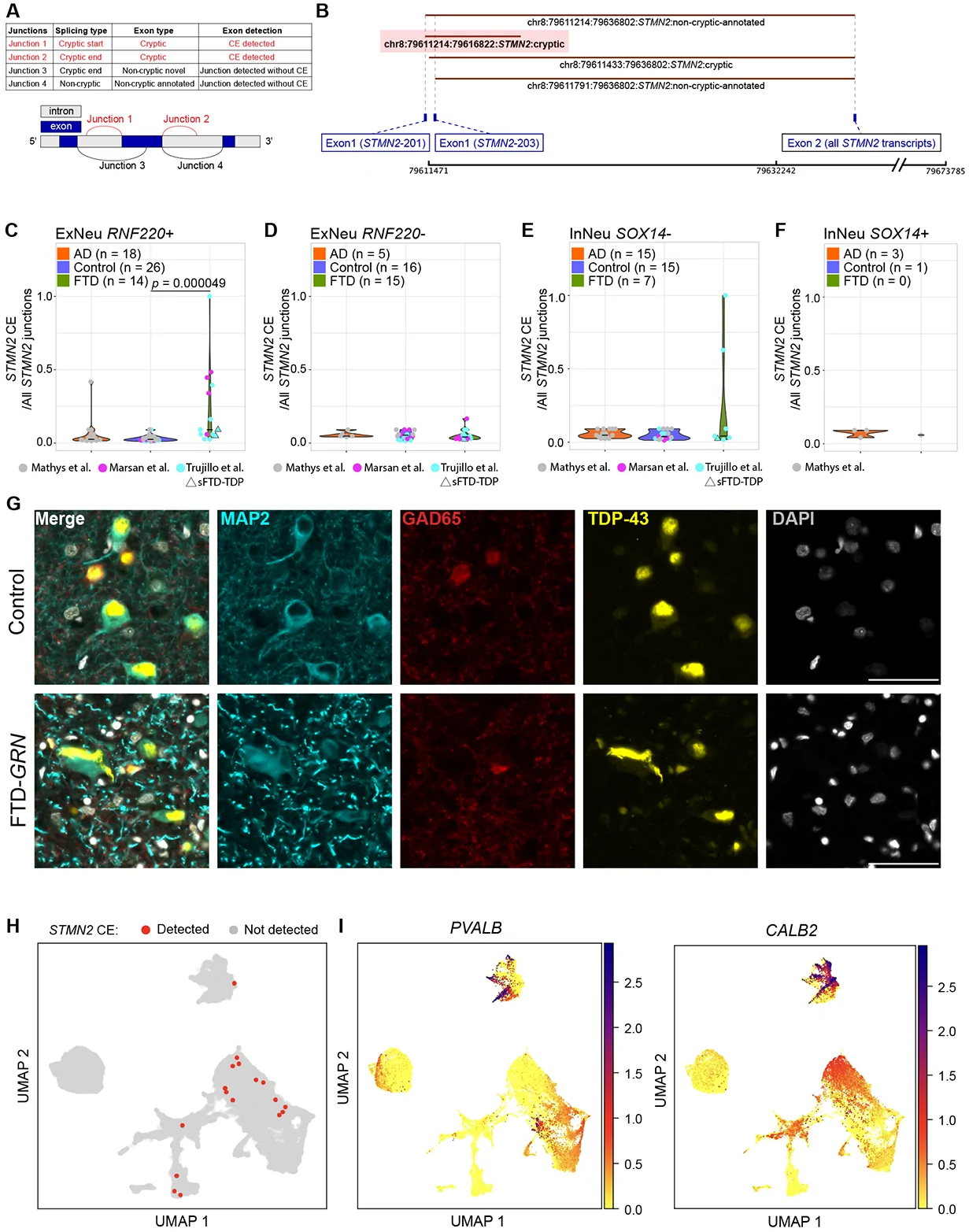
TDP-43–associated *STMN2* cryptic exon pathology in thalamic *RNF220+* excitatory neurons. (A) Schematic illustrating theoretical normal junction and cryptic exons (CE). (B) The four annotated junctions of *STMN2* across exons 1 and 2, including two normal junctions and two cryptic junctions. The known TDP43-related *STMN2* cryptic exon is indicated in bold and highlighted in red. (C-F) Violin plots quantifying *STMN2* CE abundance in thalamic neurons. Each dot represents the ratio of CE detected to all four types of junctions between Exon 1 and 2 as indicated in the schematic in (B). Triangular dot shapes represent FTD-TDP sporadic cases. Black horizontal lines within the violin plots represent the median ratio for each disease group. *p*-value were calculated using a two-sided Wilcoxon rank-sum test. (G) Immunofluorescence staining of neurons (MAP2), inhibitory neurons (GAD65), TDP-43 and nuclei (DAPI) in control and FTD-*GRN* thalamus. Scale bars = 50 μm. (H) Neuronal subtypes UMAP showing the cells for which the known TDP43-related *STMN2* cryptic exon was detected in red. (I) UMAP feature plots showing expression levels of representative core and matrix associated excitatory neuron subtypes. Color intensity represents relative gene expression (counts per million).

**Figure 5:**
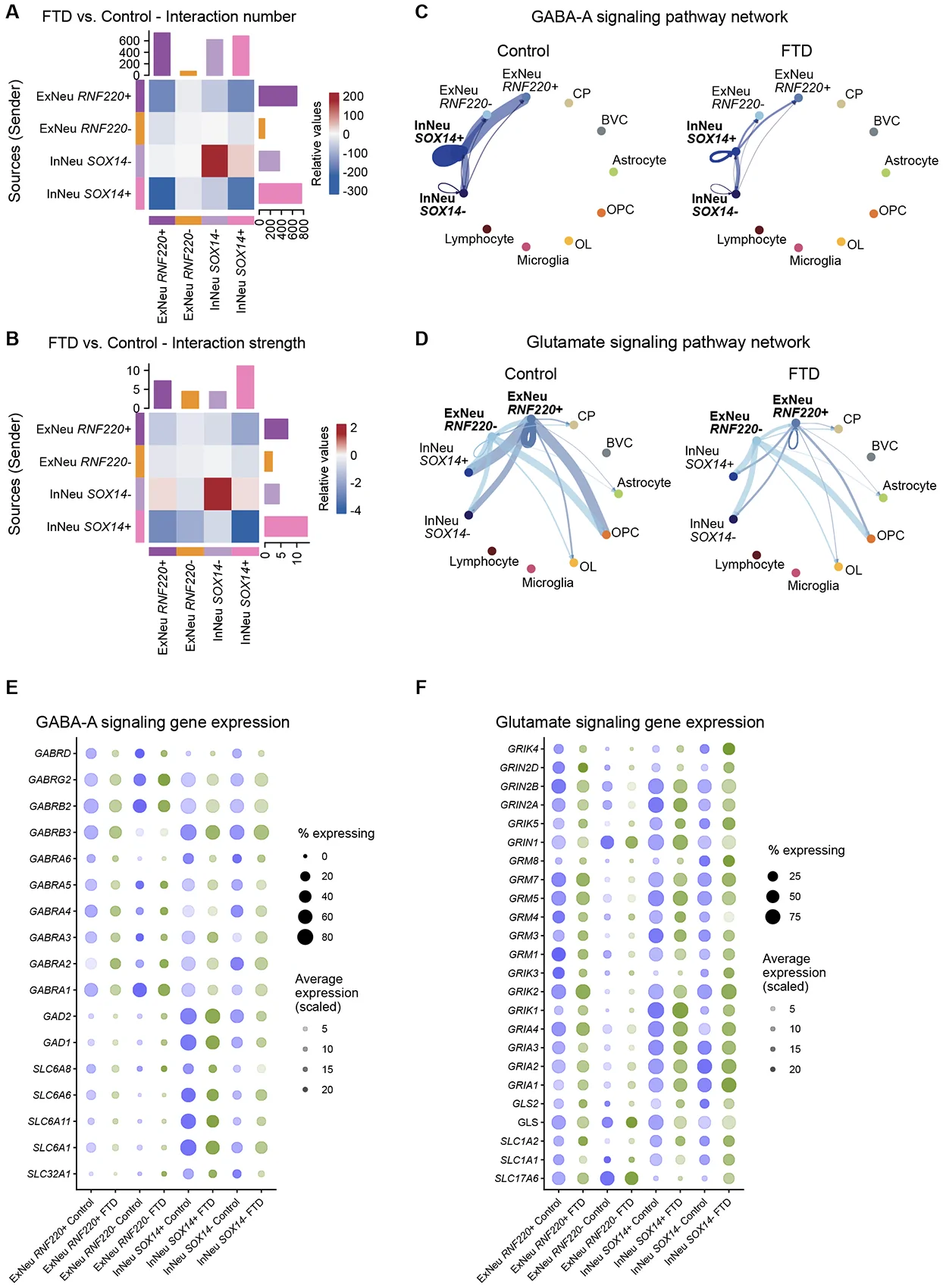
Reduced circuit connectivity in FTD. (A,B) CellChat-inferred differential cell-cell communication (A) interaction number and (B) interaction strength between thalamic neuronal subtypes. Heatmaps display relative differences between FTD cases and healthy controls from the same two studies. Red indicates increased or strengthened signaling in FTD; blue indicates decreased or weakened signaling. (C) Circular plots of inferred GABA-A signaling pathway network topology in healthy controls (left) and FTD cases (right) between inhibitory neurons and other cell types. (D) Circular plots of inferred glutamate signaling pathway network topology in healthy controls (left) and FTD cases (right) from excitatory neurons to other cell populations. Edge width is proportional to communication strength and made comparable by scaling both to the same maximum strength across the two conditions. (E, F) Dot plots depicting expression of genes involved in the (E) GABA-A and (F) glutamate signaling pathways across FTD and control populations. Dot size reflects the percentage of cells expressing each gene and color intensity reflects average scaled expression within each cell type.

**Figure 6:**
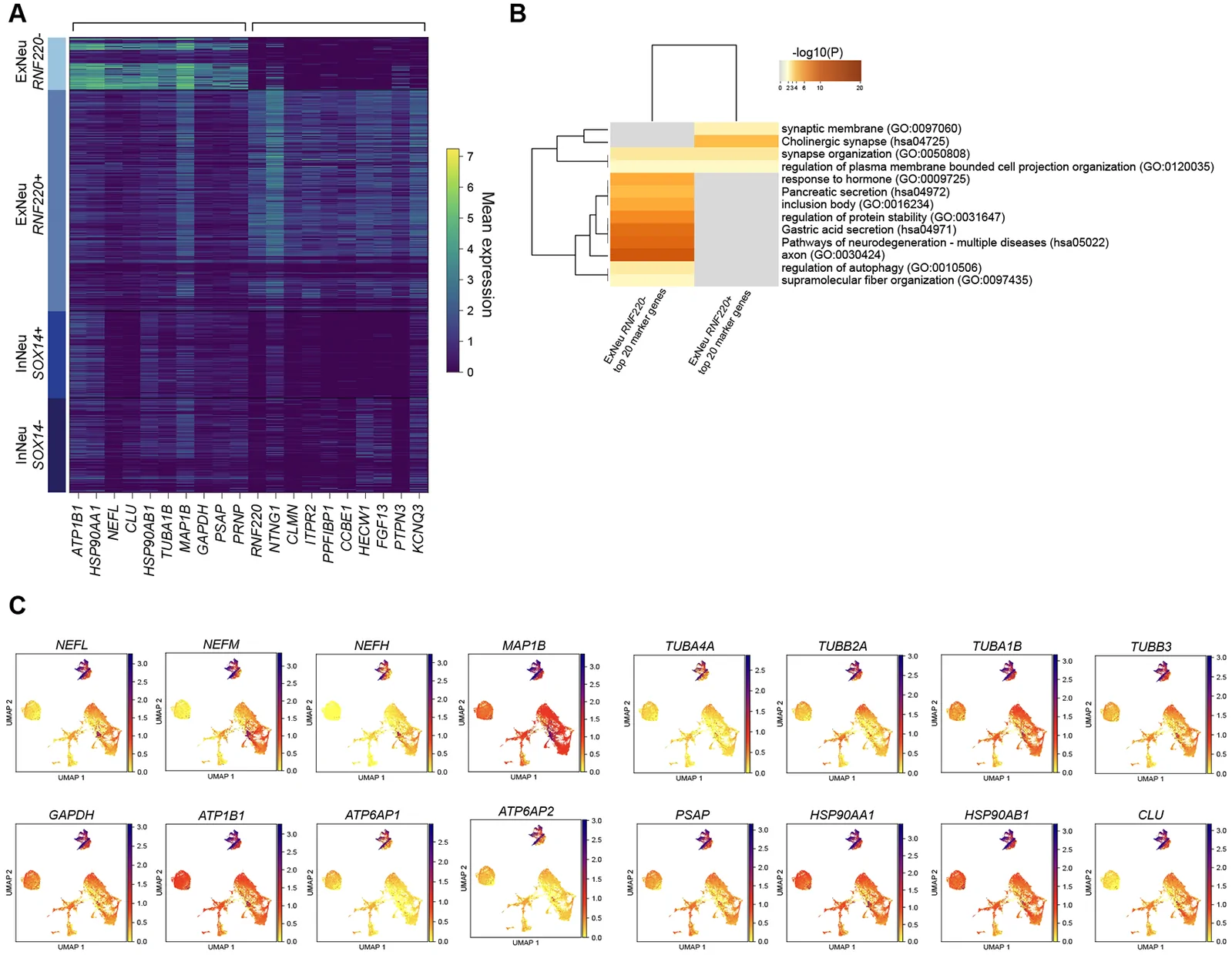
Resilience signature of *RNF220*- excitatory thalamic neurons. (A) Heatmap displaying the top 10 up- and down-regulated marker genes in ExNeu *RNF220*− (left) compared to ExNeu *RNF220*+ (right) excitatory neurons using a one versus rest Wilcoxon test. The color bar represents the mean expression of each gene (B) Metascape pathway enrichment analysis of the top 20 upregulated genes in ExNeu *RNF220*− and ExNeu *RNF220*+ populations. Color intensity represents statistical significance (-log10 P value). (C) UMAP feature plots illustrating expression levels of representative ExNeu *RNF220*− top genes across neuronal subtypes. Color intensity represents relative gene expression (counts per million).

**Figure 7:**
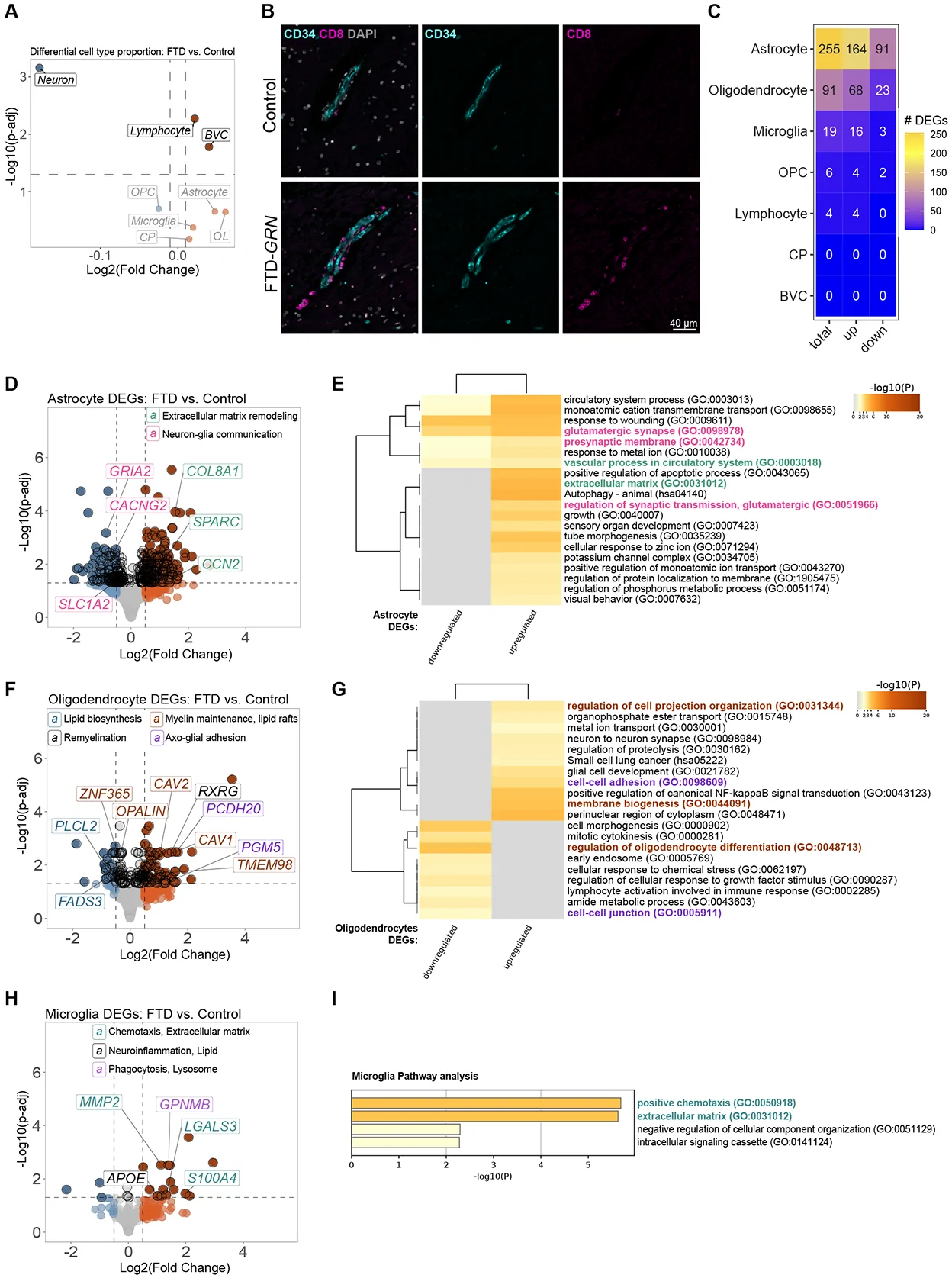
Glial cell response in FTD. (A) Volcano plot showing significant expansion of lymphocyte and BVC proportions in FTD compared to controls as estimated by differential proportion analysis. Dotted lines represent the significance cutoff (p-adj<0.05 and ∣LogFC∣>0.01). (B) Immunofluorescence staining of vascular endothelial cells (CD34), cytotoxic T cells (CD8), and nuclei (DAPI) in control and FTD-*GRN* thalamus. Scale bar = 40 μm. (C) Number of significant (padj<0.05 and ∣LogFC∣>0.5) DEGs in non neuronal cells. (D-I) Differential gene expression and functional enrichment in astrocytes and oligodendrocytes. Volcano plots displaying expression changes, with genes of interest highlighted, between FTD and Control groups (positive LogFC = Up-regulated in FTD, negative LogFC = down-regulated) in (D) astrocytes,(F) oligodendrocytes, and (H) microglia. Metascape pathway enrichment analysis of differentially up- (LogFC>0.5 and p-adj<0.05) and down-regulated (LogFC<-0.5 and p-adj<0.05) genes within (E) astrocytes, (G) oligodendrocytes, and (I) microglia. Abbreviations: OL, oligodendrocyte; OPC, oligodendrocyte precursor cell; BVC, blood vessel cell; CP, choroid plexus.

## Data Availability

10X snRNAseq FASTQ raw files for this Trujillo et al. article are available on [pending] and will be released upon publication. snRNAseq FASTQ raw files from Mathys et al. (Mathys et al., 2024) are available on synapse (syn52383413) and from Marsan et al. (Marsan et al., 2023) on SRA (PRJNA644744). 10X visium spatial transcriptomics raw files from Schulmann et al. 2026 are accessible on [pending] and will be released upon publication of that study. Processed snRNAseq and spatial transcriptomic data are available via Zenodo at [Zenodo doi].

## Code availability

Computational analyses were performed as described in the Methods section. The detailed code for individual analyses is available at https://github.com/NIH-CARD/CARD_MPU/tree/main/Thalamus_atlas.
