# Supplementary material for: Selective vulnerability and resilience of thalamic neuronal populations in Frontotemporal Dementia
Source: Res Sq. 2026 Sep 11:rs.3.rs-10670822. Preprint. [Version 1] doi: 10.21203/rs.3.rs-10670822/v1 (PMC13622835; doi:10.21203/rs.3.rs-10670822/v1)
Supplement: Supplementary file 1 [file NIHPPrs10670822v1-supplement-1.pdf]

Supplementary figures:

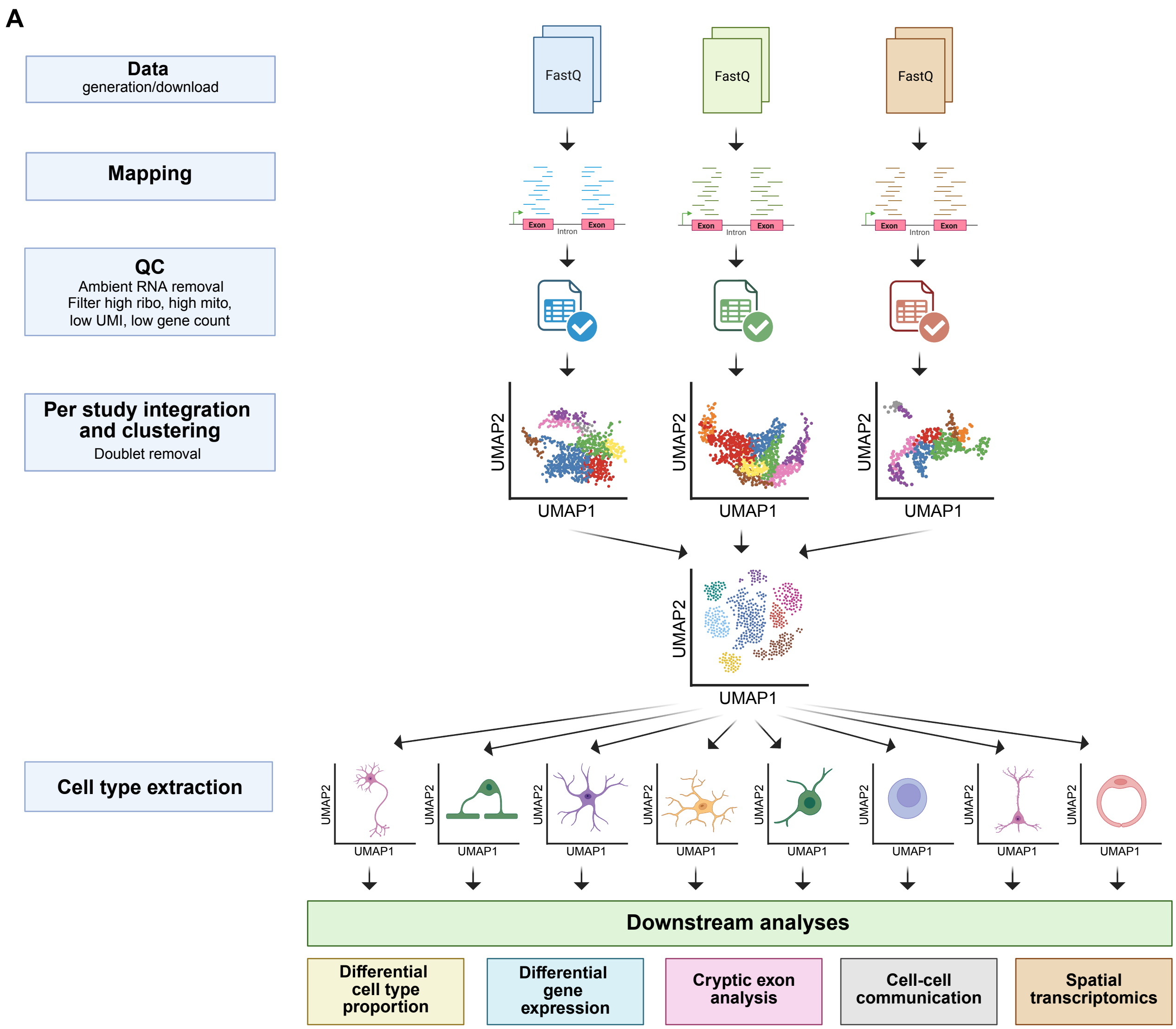

Supplementary Figure 1: snRNAseq analysis pipeline. Single nuclei RNAseq and analysis pipeline including data acquisition, pre-processing, per-study integration, atlas integration and annotation, cell type extraction, and downstream analyses. Schematic was created with BioRender (BioRender.com).

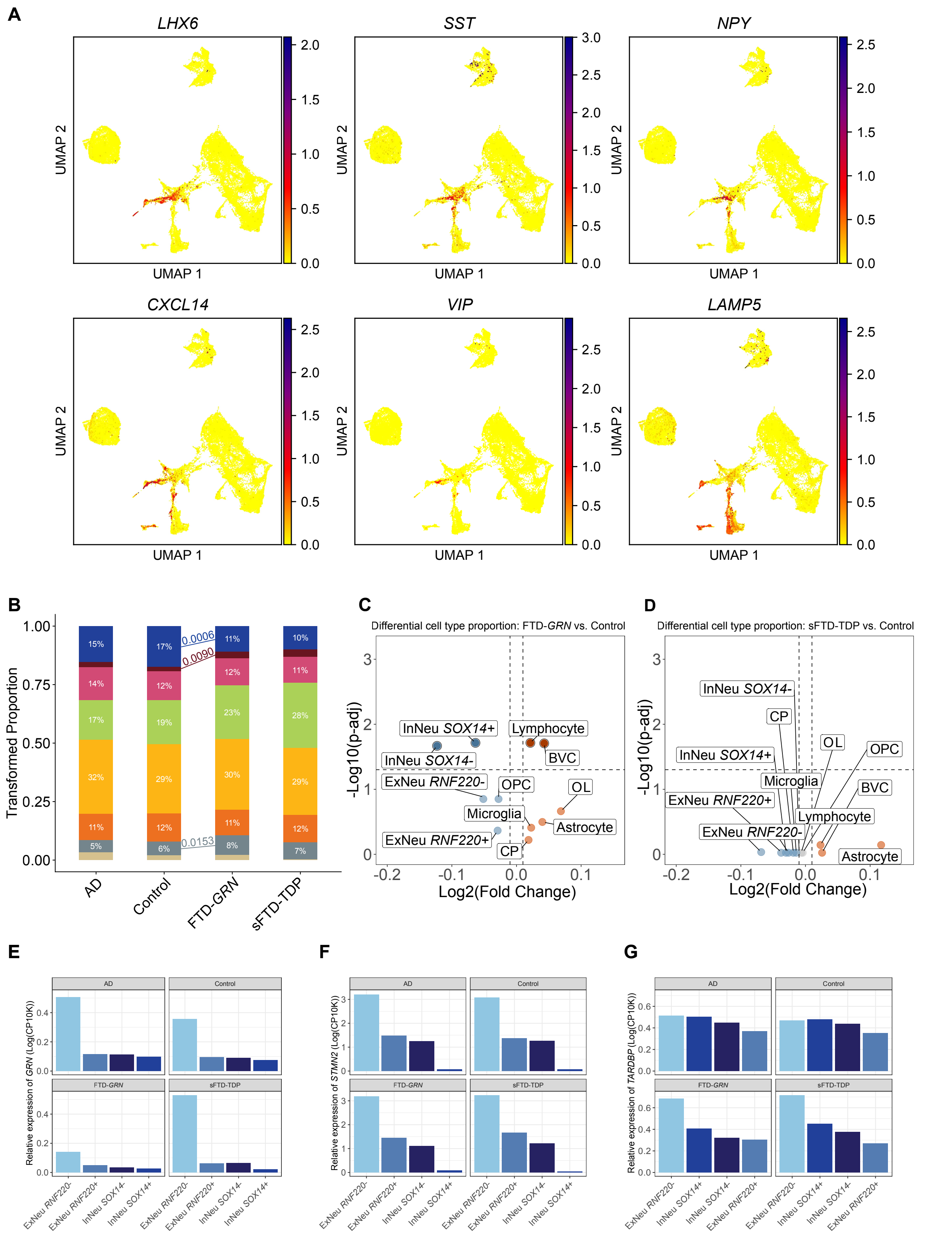

Supplementary Figure 2: Differential cell type proportion in FTD-*GRN* vs. control and sFTD-TDP vs. control. (A) UMAP feature plots showing expression levels of representative ganglionic

eminence-associated inhibitory neuron lineages and subtypes. Color intensity represents relative gene expression (counts per million) (B) Bar plot displaying transformed cell type proportions across control, AD, FTD-*GRN*, and sFTD-TDP disease status, with significant adjusted p-values from differential cell type proportion analysis shown for neurons, lymphocytes, and BVC. Adjusted p-values were calculated by linear model using limma's lmFit and moderated using eBayes. (B-C) Volcano plot showing the differential cell type proportion across the brain cell types, including the four thalamic neuronal subtypes, where dotted lines represent the significance cutoff (p-adj<0.05 and |LogFC|>0.01). In (C) FTD-*GRN* vs. Control, and (D) sFTD-TDP vs. control. Abbreviations: OL, oligodendrocyte; OPC, oligodendrocyte precursor cell; BVC, blood vessel cell; CP, choroid plexus. (E-G) relative Log(CP10K) expression in neuronal subtypes between AD, Control, FTD-*GRN*, and sFTD-TDP for (E) *GRN*, (F) *STMN2*, and (G) *TARDBP*.

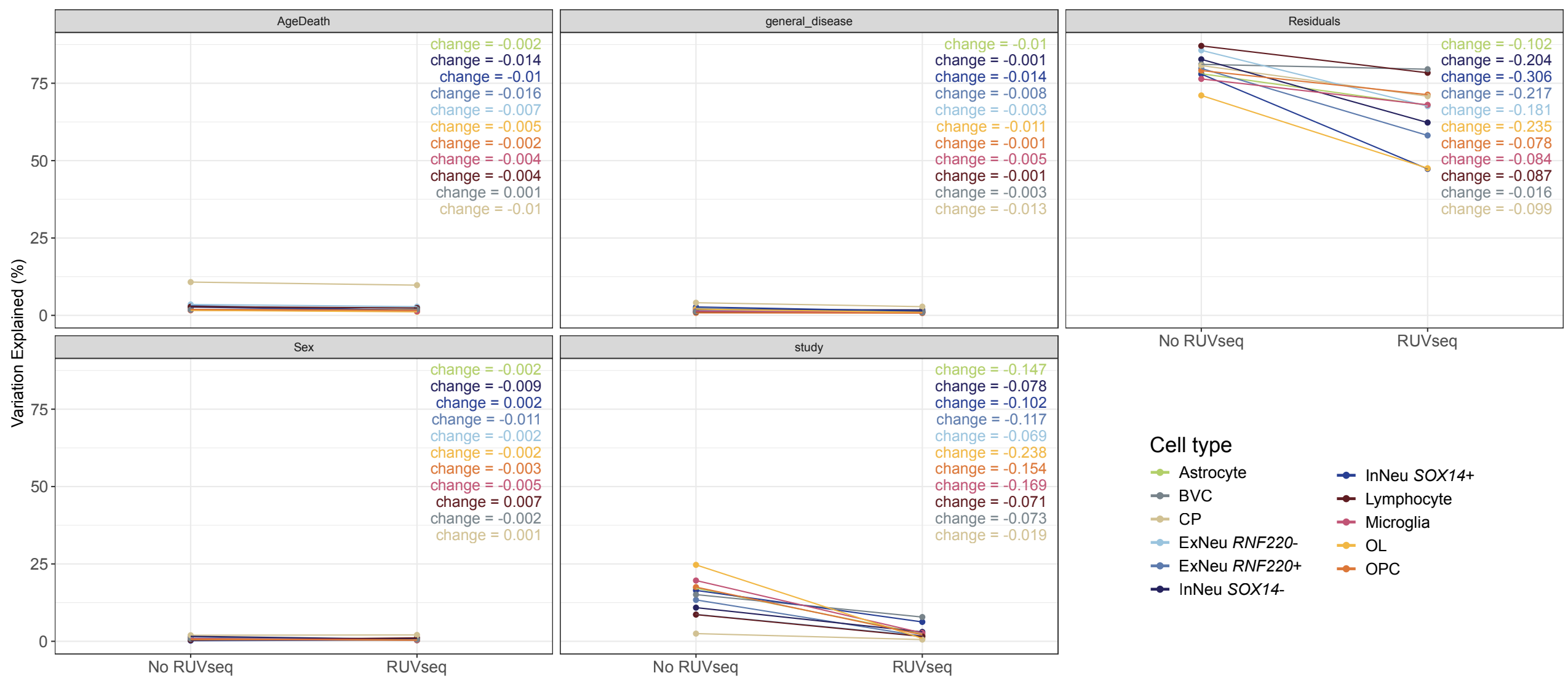

Supplementary Figure 3: RUVseq impact on explained variance. A paired line-plot depicting the proportion of variance explained before and after running RUVseq correction in each cell type for each covariate and residuals. Each line represents a cell type, and values indicate the change in variance explained following correction. Abbreviations: OL, oligodendrocyte; OPC, oligodendrocyte precursor cell; BVC, blood vessel cell; CP, choroid plexus.

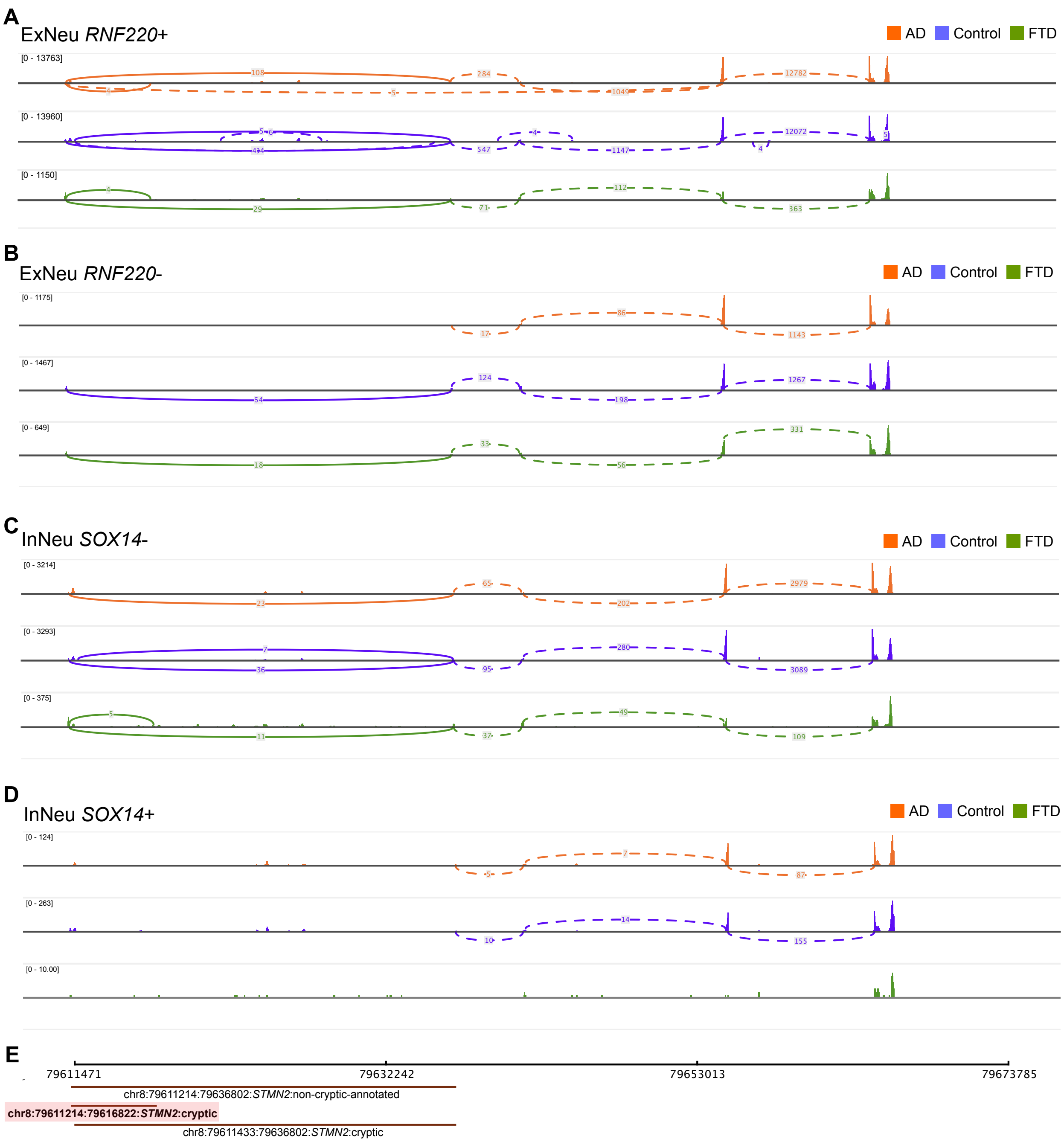

Supplementary Figure 4: *STMN2* splice junctions and expression of *STMN2* and *TARDBP*. (A-D) Sashimi plots illustrating *STMN2* cryptic exon inclusion across the four thalamic neuron subtypes in FTD-*GRN*. Individual arches represent splice junctions with a minimum raw count of 4. Solid arches represent splice junctions within a junction cluster, computed using LeafCutter, where junctions share either a start or end genomic coordinate. Dashed arches represent splice junctions not retained in the current junction cluster. Numbers embedded in individual arches indicate raw read counts for the corresponding splice junctions. In the schematic, brown horizontal lines indicate genomic coordinates and CE labels for the individual splice junctions represented by the solid arches. The CE label in bold represents the known target of TDP43. (A) ExNeu *RNF220+*, (B) ExNeu *RNF220-*, (C) InNeu *SOX14-*, and (D) InNeu *SOX14+*. (E) Reference genome annotation coordinates

of *STMN2* gene. Annotated junctions of *STMN2* across exons 1 and 2, including two normal junctions and two cryptic junctions. The known TDP43-related *STMN2* cryptic exon is indicated in bold and highlighted in red.

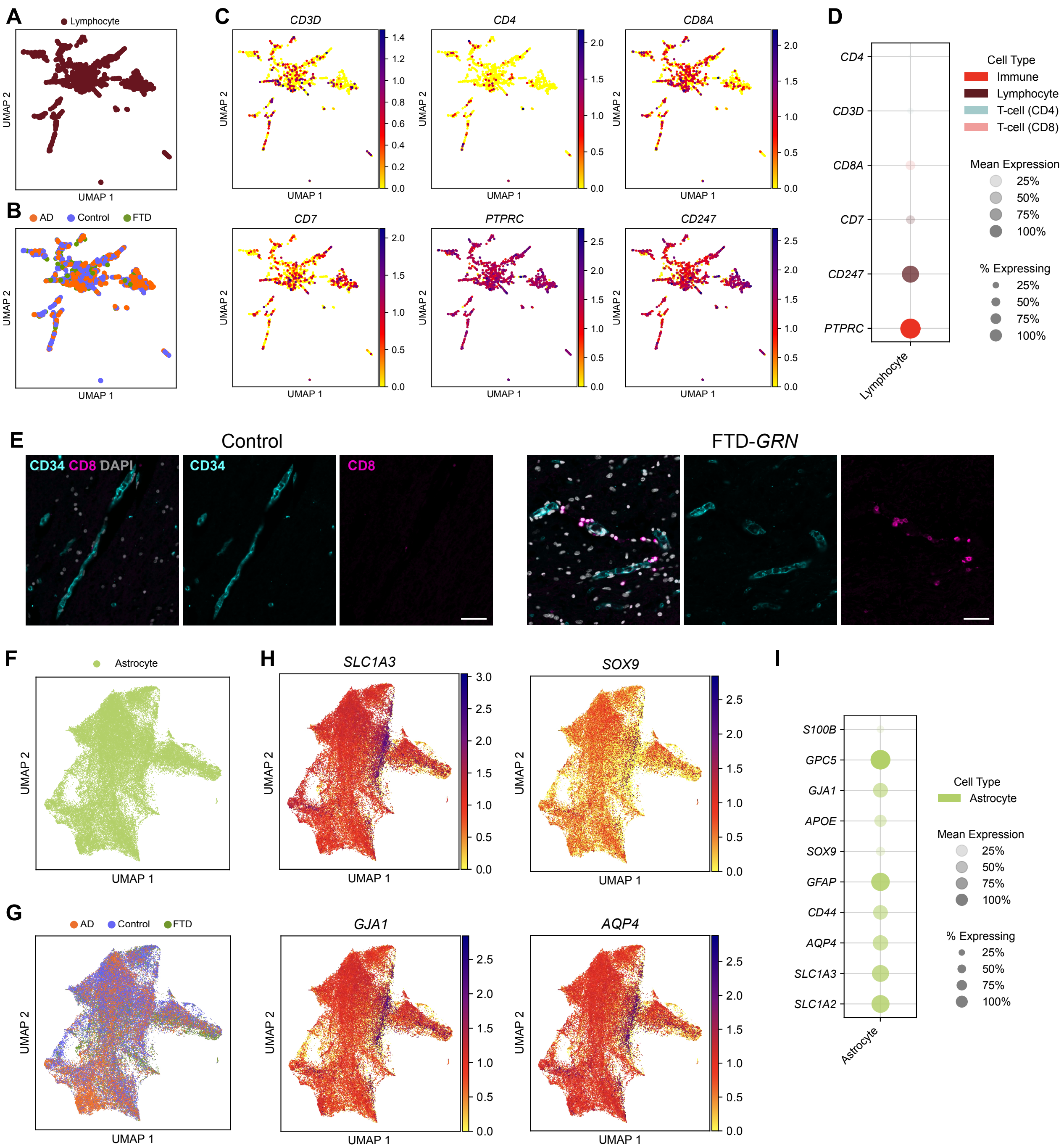

Supplementary Figure 5: Expression of marker genes for lymphocytes and astrocytes, and endothelial cells and cytotoxic T cells staining. (A) UMAP of the lymphocyte cluster. (B) UMAP projection colored by disease condition. (C) UMAP feature plots showing expression levels of marker genes defining lymphocytes subtypes. Color intensity represents relative gene expression (counts per million). (D) Dot plot of marker genes defining lymphocyte subtypes where dot size reflects the proportion of cells expressing each gene and color intensity reflects mean expression within each cell type. (E) Immunofluorescence staining of vascular endothelial cells (CD34), cytotoxic T cells (CD8), and nuclei (DAPI) in control and FTD-*GRN* thalamus. Scale bars = 40 µm. (F) UMAP of the astrocyte cluster. (G) UMAP projection colored by disease condition. (H) UMAP feature plots showing the expression level of marker genes expression defining astrocytes. Color intensity

represents relative gene expression. (I) Dot plot of marker genes defining astrocyte sub-populations where dot size reflects the proportion of cells expressing each gene and color intensity reflects mean expression within each cell type.

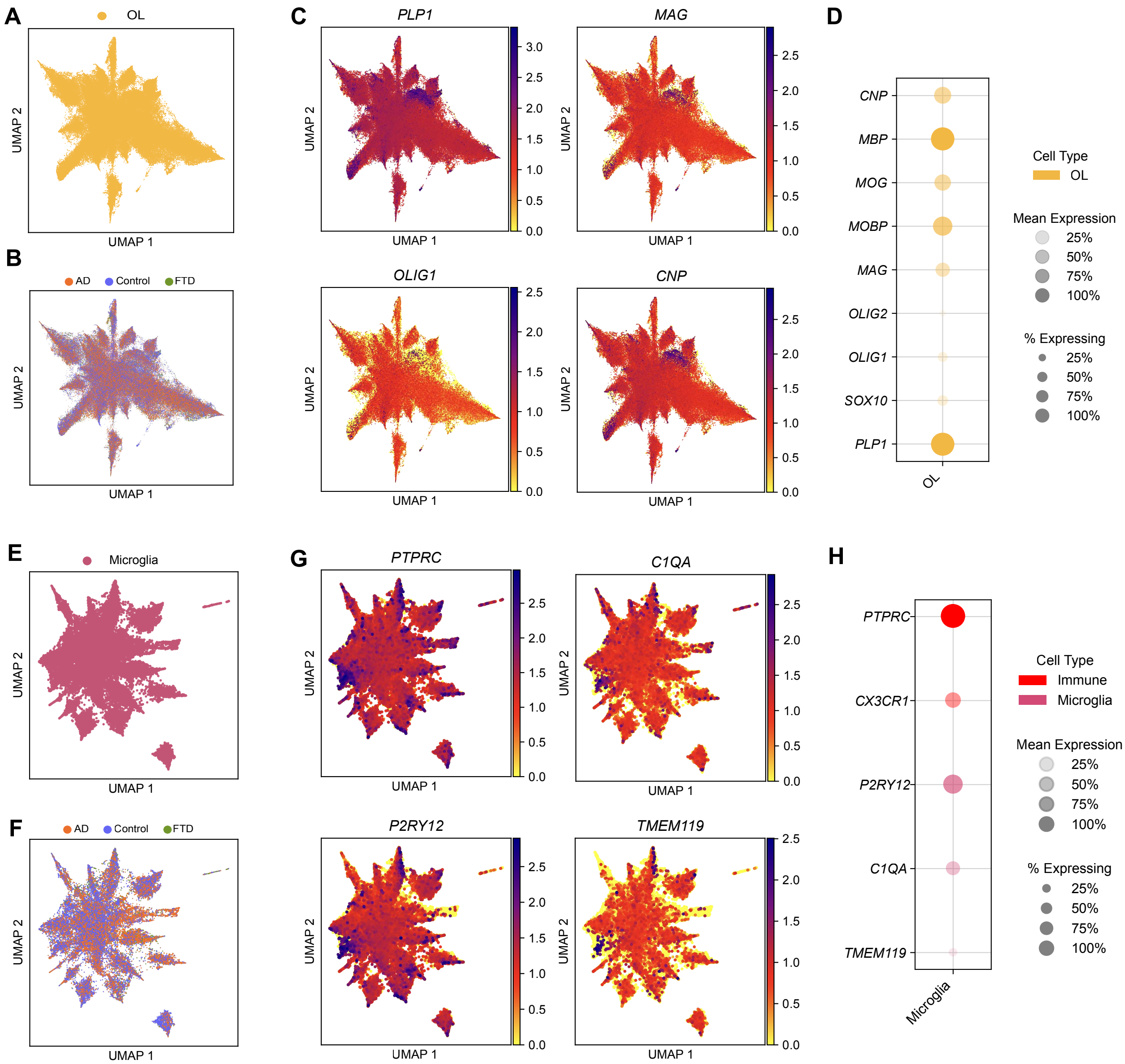

Supplementary Figure 6: Expression of marker genes for oligodendrocytes and microglia. (A) UMAP of the oligodendrocyte (OL) cluster. (B) UMAP projection colored by disease condition. (C) UMAP feature plots showing the expression level of marker genes defining oligodendrocytes. Color intensity represents relative gene expression. (D) Dot plot of marker genes defining oligodendrocytes where dot size reflects the proportion of cells expressing each gene and color intensity reflects mean expression within each cell type. (E) UMAP of the microglia cluster. (F) UMAP projection colored by disease condition. (G) UMAP feature plots representing the expression levels of marker genes defining microglia. Color intensity represents relative gene expression. (H) Dot plot of marker genes defining microglia where dot size reflects the proportion of cells expressing each gene and color intensity reflects mean expression within each cell type. Abbreviation: OL, oligodendrocyte.
